# Efficient Triplet‐Triplet Annihilation Upconversion Sensitized by a Chromium(III) Complex via an Underexplored Energy Transfer Mechanism

**DOI:** 10.1002/anie.202202238

**Published:** 2022-05-09

**Authors:** Cui Wang, Florian Reichenauer, Winald R. Kitzmann, Christoph Kerzig, Katja Heinze, Ute Resch‐Genger

**Affiliations:** ^1^ Division Biophotonics Federal Institute for Materials Research and Testing (BAM) Richard-Willstätter-Strasse 11 12489 Berlin Germany; ^2^ Institute of Chemistry and Biochemistry Free University of Berlin Arnimallee 22 14195 Berlin Germany; ^3^ Department of Chemistry Johannes Gutenberg University of Mainz Duesbergweg 10–14 55128 Mainz Germany

**Keywords:** Chromium, Dexter Energy Transfer, Doublet-Triplet Energy Transfer, Triplet-Triplet Annihilation, Upconversion

## Abstract

Sensitized triplet‐triplet annihilation upconversion (sTTA‐UC) mainly relies on precious metal complexes thanks to their high intersystem crossing (ISC) efficiencies, excited state energies, and lifetimes, while complexes of abundant first‐row transition metals are only rarely utilized and with often moderate UC quantum yields. [Cr(bpmp)_2_]^3+^ (bpmp=2,6‐bis(2‐pyridylmethyl)pyridine) containing earth‐abundant chromium possesses an absorption band suitable for green light excitation, a doublet excited state energy matching the triplet energy of 9,10‐diphenyl anthracene (DPA), a close to millisecond excited state lifetime, and high photostability. Combined ISC and doublet‐triplet energy transfer from excited [Cr(bpmp)_2_]^3+^ to DPA gives ^3^DPA with close‐to‐unity quantum yield. TTA of ^3^DPA furnishes green‐to‐blue UC with a quantum yield of 12.0 % (close to the theoretical maximum). Sterically less‐hindered anthracenes undergo a [4+4] cycloaddition with [Cr(bpmp)_2_]^3+^ and green light.

## Graphical abstract

The molecular ruby [Cr(bpmp)_2_]^3+^ with an earth‐abundant chromium(III) central ion sensitizes green‐to‐blue triplet‐triplet annihilation upconversion with 9,10‐diphenylanthracene as the annihilator. The process involves a near‐unity energy transfer efficiency via an underexplored reaction pathway, an anti‐Stokes shift of 0.54 eV, and a maximum upconversion quantum yield of 12.0 %.

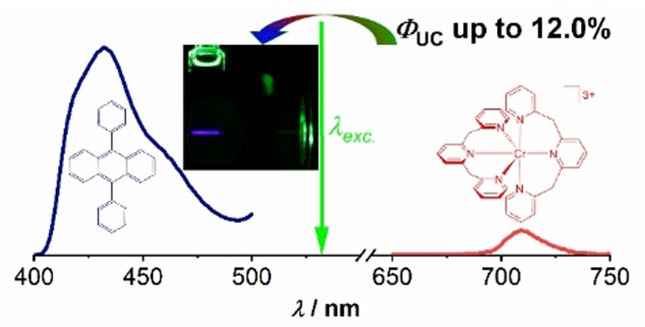

## Introduction

Triplet‐triplet annihilation‐based sensitized photon upconversion (sTTA‐UC) converts low‐energy photons into anti‐Stokes‐shifted photons or allows demanding photochemical activation of substrates with low‐energy light.^[1]^ Typically, a sTTA‐UC system consists of a strongly absorbing sensitizer with a long‐lived excited triplet state and a highly fluorescent organic annihilator. Photoexcitation of the sensitizer, which is often a transition metal complex with a precious metal, from its singlet ground state to a singlet excited state (frequently a singlet metal‐to‐ligand charge‐transfer state ^1^MLCT), is followed by intersystem crossing (ISC) to populate its long‐lived triplet state (often a ^3^MLCT state). The electronically excited triplet photosensitizer engages in collisional triplet‐triplet energy transfer (TTET) via a Dexter energy transfer mechanism with an acceptor possessing an appropriate triplet (T_1_) energy. Interaction of two triplet excited acceptors leads to triplet‐triplet annihilation (TTA), which yields one acceptor in its ground state S_0_ and the second one in its excited singlet state S_1_. The latter releases its energy by emitting delayed fluorescence at a shorter wavelength (higher energy) than the photons originally absorbed by the sensitizer.[^1a^, ^1b^, ^1c^, ^1d^, ^1e^, ^1f^, ^1g^, ^1h^, ^1i^] Alternatively, this S_1_ state can be used for chemical transformations requiring more energy than a single low‐energy photon.[^1j^, ^1k^, ^1l^, ^1m^, ^1n^, ^1o^]

(1) $$\Phi_{UC}=\frac{f}{2}\Phi_{ISC}\Phi_{EnT}\Phi_{TTA}\Phi_{F}$$

Equation 1^[2]^ describes the dependence of the upconversion quantum yield (*Φ*_UC_) on the efficiencies of the involved processes, i.e., ISC of the sensitizer (*Φ*_ISC_), energy transfer (EnT; often TTET from ^3^MLCT states) from the excited sensitizer to the annihilator (*Φ*_EnT_), TTA of the annihilator (*Φ*_TTA_), and delayed annihilator fluorescence (*Φ*_F_). *f* equals the spin‐statistical factor for generating a singlet spin‐state via TTA. The combination of two acceptors in their T_1_ states in the TTA encounter complex generates nine spin states, namely one singlet, three triplet, and five quintet states. Depending on the energies of the annihilator's T_1_, T_2_, and S_1_ states, the theoretical maximum of the spin‐statistical factor *f*_max_ amounts to 11.1 %, 40 % or 100 % for all channels (2*E*(T_1_)>*E*(S_1_); usually not accessible due to the too high energy of the quintet state), only singlet and triplet channels (2*E*(T_1_)>*E*(S_1_) and 2*E*(T_1_)>*E*(T_2_)), or only the singlet channel (*E*(T_2_)>2*E*(T_1_)>*E*(S_1_)) being open.^[3]^ This leads to maximum UC quantum yields (*Φ*_UC,max_) of 5.55 %, 20 % or 50 %, respectively, for *Φ*_ISC_, *Φ*_EnT_, *Φ*_TTA_, and *Φ*_F_ amounting to 100 %, taking the reaction stoichiometry into account.[^2a^, ^4^] The excited states of the most often used blue fluorescent annihilator 9,10‐diphenyl anthracene (DPA) allow both open singlet and triplet channels (*Φ*_UC,max_=20 % for *f*_max_=40 %).[^3a^, ^5^] Occasionally, *Φ*_UC_ values >20 % were observed as well,^[6]^ as other factors like spin dynamics and shapes of the excited singlet and triplet energy surfaces of the annihilator can also affect the overall UC efficiency.[^3a^, ^7^]

From the sensitizer perspective, both ISC and energy transfer—in most cases TTET—can be optimized. The ISC process is greatly accelerated in sensitizers with heavy metal ions, such as [Ru(bpy)_3_]^2+^ (bpy=2,2′‐bipyridine) and its derivatives. This explains the ubiquitous utilization of noble metal complexes in sTTA‐UC and other photochemical applications. Energy transfer processes from the excited sensitizer to the annihilator via the Dexter mechanism^[8]^ require an overlap of the excited state wavefunction of the sensitizer and the annihilator's ground state wavefunction. Consequently, (triplet) charge‐transfer excited states with a wavefunction extending onto the ligands are particularly well suited for Dexter energy transfer. This lays the basis for exploiting complexes such as [Ru(bpy)_3_]^2+^ and its derivatives with long‐lived charge‐transfer states (mostly ^3^MLCT) in UC processes.^[1h]^

Most molecular triplet sensitizers featuring long‐lived excited charge‐transfer states contain precious second‐ or third‐row transition metals, e.g. Ru^II^, Ir^III^, Re^I^, Pt^II^, Pd^II^ or Os^II^.^[9]^ Replacing precious metals with more earth‐abundant transition metals, in particular first‐row metals, opens non‐radiative decay pathways via low‐energy metal‐centered (MC) excited states.^[10]^ This can reduce their excited state lifetimes. Notable exceptions are Cu^I 3^MLCT and Zr^IV 3^LMCT sensitizers with 3d^10^ and 4d^0^ electron configurations that lack detrimental MC excited states,[^6d^, ^11^] as well as Cr^0^, Mn^I^, and Mo^0^ triplet sensitizers with a d^6^ electron configuration in a strong ligand field.[^1l^, ^12^]

Fundamentally different from long‐lived ^3^MLCT (or ^3^LMCT) excited states are intraconfigurational MC excited states which are characterized by a spin‐flip within the lower‐energy d orbitals (t_2g_ orbitals in octahedral symmetry).^[13]^ Thanks to the spin‐forbidden and often Laporte‐forbidden character of these spin‐flip transitions, the excited state lifetimes can even reach milliseconds.[^13a^, ^14^] Certain Cr^III^ complexes are called “molecular rubies” owing to their excited state landscapes resembling that of the oxidic mineral ruby, and they possess extraordinarily long excited state lifetimes and record photoluminescence quantum yields.[^13a^, ^14^] However, their excited state energies are very low.[^13a^, ^14a^, ^14b^, ^14c^, ^14d^, ^14f^, ^15^] This precludes their utilization in sTTA‐UC due to the mismatch with the triplet energies of typical annihilators like DPA.^[1h]^ Moreover, their electronic structure renders efficient excitation at wavelength >470 nm barely feasible. For these Cr^III^ complexes, energy transfer to triplet oxygen[^14e^, ^16^] or to lanthanide ions with low energy excited states has been successfully demonstrated as well as UC luminescence (UCL) using Cr^III^→Er^III^ energy transfer.^[17]^ Energy transfer from Yb^III^ to Cr^III^ has also been reported to yield NIR‐to‐NIR upconverted photons.^[18]^ Energy transfer from the π‐π* states of appended anthracenyl substituents to Cr^III^ leads to quenching of the anthracene fluorescence and sensitized phosphorescence from the metal‐centered doublet state.^[19]^ The reverse process of sensitizing the triplet state of anthracenes or other organic dyes by electronically excited Cr^III^ complexes (doublet‐triplet energy transfer, DTET), relevant for sTTA‐UC, has not been observed so far due to the lack of Cr^III^ complexes with suitably high doublet state energies and long excited state lifetimes.[^13a^, ^14a^, ^14b^, ^14c^, ^14d^, ^15^] The recently reported second generation molecular ruby [Cr(bpmp)_2_]^3+^ has a comparably high doublet energy (1.75 eV) and a high excited spin‐flip state lifetime in the low ms range (e.g., 1550 μs in H_2_O/HClO_4_) along with a comparably low‐energy absorption band at 462 nm (bpmp=2,6‐bis(2‐pyridylmethyl)pyridine).^[14e]^ This bands tails up to about 550 nm, enabling excitation with green light. These photophysical properties recommend [Cr(bpmp)_2_]^3+^ as potential sensitizer in sTTA‐UC schemes.

We now report on unprecedented UC and photochemical cycloaddition reactions using the [Cr(bpmp)_2_]^3+^ sensitizer and green light excitation. The unique decisive DTET process from the excited Cr^III^ complex to anthracene acceptors is probed by steady‐state and time‐resolved photoluminescence spectroscopy and excitation power density (*P*) dependent luminescence studies, complemented by nanosecond transient absorption (TA) spectroscopy.

## Results and Discussion

**Figure 1:**
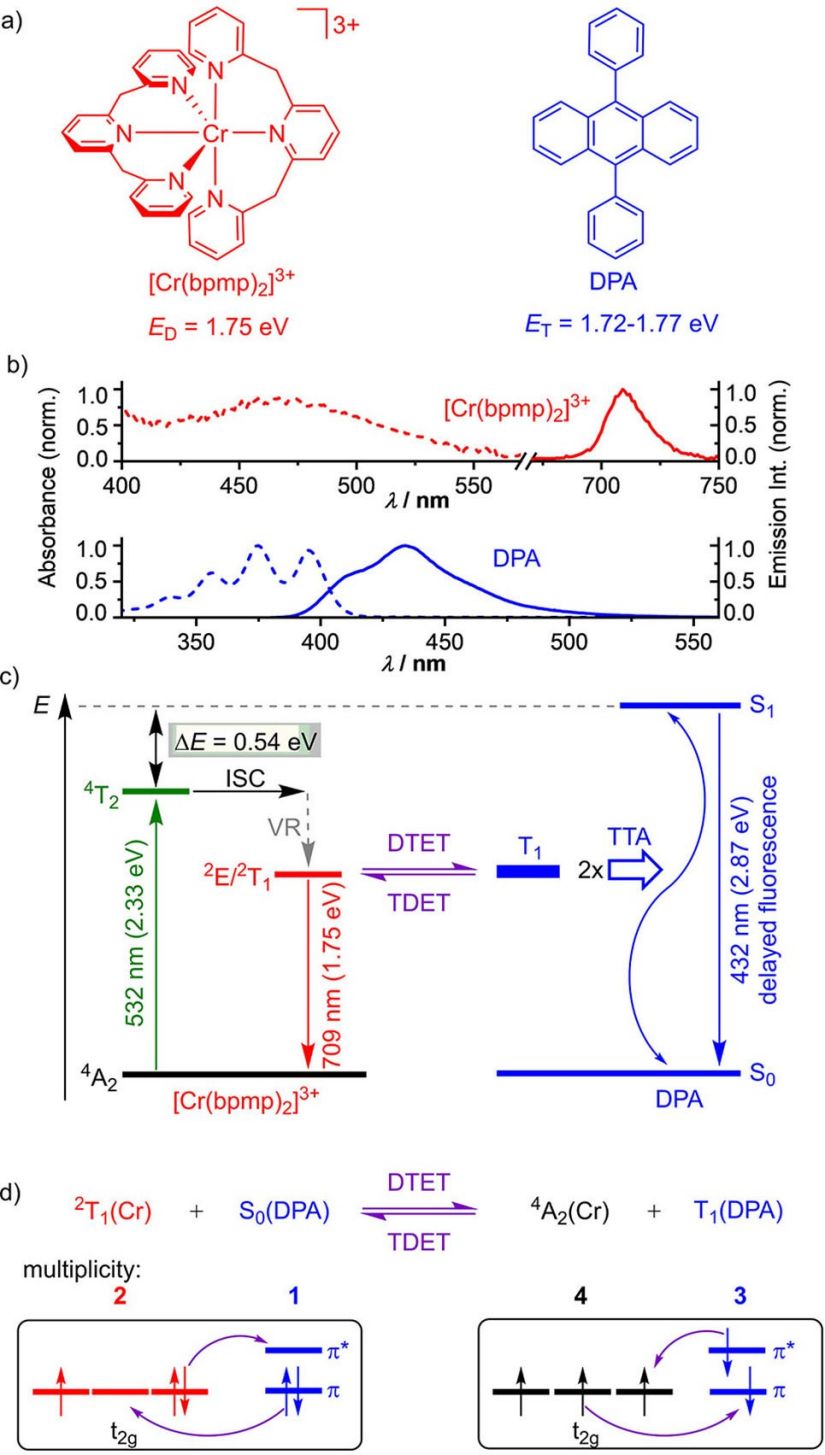
a) Structures and excited state energies of [Cr(bpmp)_2_]^3+^ and DPA, b) normalized absorption and emission spectra of [Cr(bpmp)_2_]^3+^ and DPA, c) Jablonski diagram illustrating the excitation of the sensitizer with green light, intersystem crossing (ISC) to its doublet states, vibrational relaxation (VR), doublet‐triplet energy transfer (DTET), the reverse process triplet‐doublet energy transfer (TDET), triplet‐triplet annihilation (TTA) and delayed fluorescence, and d) illustration of the Dexter energy transfer between the excited [Cr(bpmp)_2_]^3+^ sensitizer and ground state DPA (DTET) and the reverse process TDET using relevant microstates. The multiplicities (spin degeneracies) of the involved states are highlighted.

The key idea is to identify a suitable Cr^III^ based sensitizer which meets the sTTA‐UC requirements of a low excitation energy, a doublet excited state energy matching the energy of the DPA annihilator in its triplet state T_1_ (*E*_T_=1.72–1.77 eV; Figure 1a),[^3a^, ^20^] and a long excited state lifetime. To the best of our knowledge, out of the huge number of known Cr^III^ complexes, only the Cr^III^ complex [Cr(bpmp)_2_]^3+^ fulfills these requirements. [Cr(bpmp)_2_]^3+^ has an excitation energy of 2.33 eV (532 nm), a doublet excited state (^2^E/^2^T_1_) energy of 1.75 eV, an excited state lifetime of *τ*_0_=890 μs independent of concentration (in deoxygenated DMF containing 0.1 M perchloric acid), and a high photostability (Figures 1a and 1b; Supporting Information, Figures S1, S2).^[14e]^

According to the intensity‐ and lifetime‐based Stern–Volmer studies, DPA quenches the [Cr(bpmp)_2_]^3+^ phosphorescence at 709 nm with *K*_SV_=5.7×10^4^ M^−1^ and 5.0×10^4^ M^−1^, respectively (Figure S3). The similar *K*_SV_ values confirm dynamic quenching^[21]^ of the ^2^E/^2^T_1_ states of [Cr(bpmp)_2_]^3+^ via DTET to DPA (Figure 1c)). The DTET rate constant *k*_DTET_=*K*_SV_/*τ*_0_ amounts to (5.6–6.4)×10^7^ M^−1^ s^−1^. This value is lower than the diffusion limit of bimolecular reactions in DMF (830×10^7^ M^−1^ s^−1^) at 25 °C^[22]^ but similar to the rates of self‐exchange reactions of Cr^III^ complexes involving doublet‐doublet energy transfer.^[23]^

To visualize possible encounters of [Cr(bpmp)_2_]^3+^ and DPA and to estimate the distances involved in the Dexter‐type DTET processes with Cr^III^ sensitizers, molecular models of anthracene as small DPA model and [Cr(bpmp)_2_]^3+^ in a solvent cage were optimized with Density Functional Theory calculations (CPCM(DMF)‐RI‐B3LYP‐D3BJ‐ZORA/def2‐TZVPP; Figure S4). These qualitative models, which do not consider counter ions and solvent molecules, do not support a strong and static interaction between [Cr(bpmp)_2_]^3+^ and anthracene in agreement with the dynamic quenching of the doublet states. The models of the exemplary optimized collisional encounter complexes suggest shortest distances of 5.8–9.5 Å between the Cr^III^ ions to the centers of the anthracene rings. These distances are compatible with a Dexter mechanism^[8]^ of the DTET process (Figure S4). Compared to sensitizers with triplet charge‐transfer states,[^1l^, ^6d^, ^9^, ^10^, ^11^, ^12^] where the wavefunctions of the excited states extend onto the ligands, the metal‐localized nature of the Cr^III^ spin‐flip states^[13]^ allows only for comparably weak sensitizer‐annihilator orbital interactions in the encounter complexes. This—in addition to the small driving force for DTET (Figure 1c)—can explain the roughly 4.4–5.0 times lower energy transfer rate constant of [Cr(bpmp)_2_]^3+^/DPA as compared to, e.g. the triplet‐triplet energy transfer (TTET) rate constant *k*_TTET_ of 28.2×10^7^ M^−1^ s^−1^ reported by Castellano et al. for DPA and a Cu^I^‐based ^3^MLCT sensitizer.^[24]^

**Figure 2:**
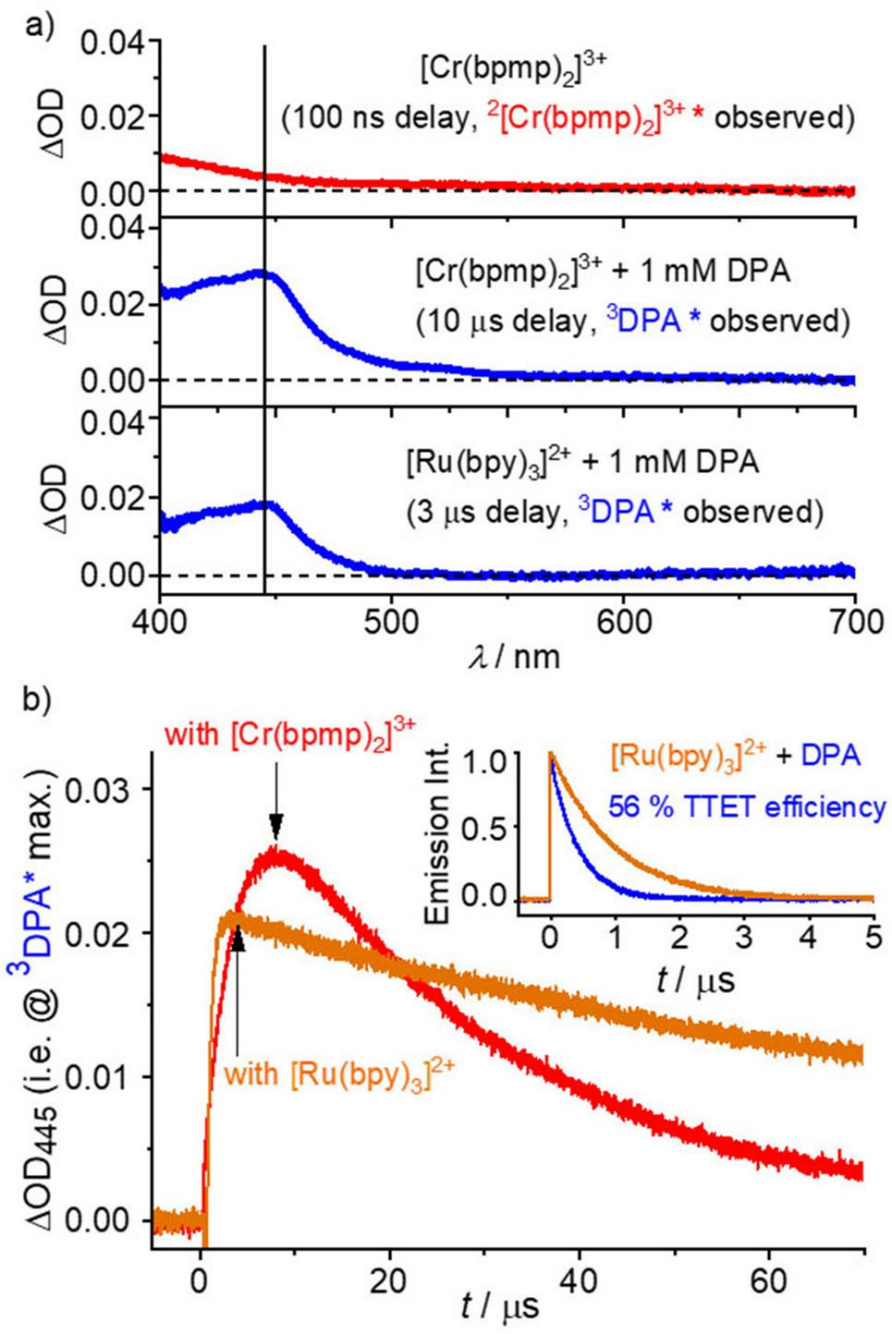
Transient absorption studies of the formation of ^3^DPA upon sensitizer excitation with a 532 nm laser in Ar‐saturated acidified DMF containing 1 mM of DPA. a) TA spectra of [Cr(bpmp)_2_]^3+^ (middle panel) and [Ru(bpy)_3_]^2+^ (bottom panel) in the presence of DPA integrated over 100 ns as well as control experiments with [Cr(bpmp)_2_]^3+^ (upper panel). b) Comparative TA traces monitoring the formation and decay of ^3^DPA with [Cr(bpmp)_2_]^3+^ and [Ru(bpy)_3_]^2+^ under these conditions. The measurement of the ^3^[Ru(bpy)_3_]^2+^ TTET quenching efficiency is shown in the inset. The concentrations of [Cr(bpmp)_2_]^3+^ and [Ru(bpy)_3_]^2+^ were adjusted to matching absorbances of 0.027±0.001 at 532 nm. The detection windows used for the measurement of the ^3^DPA spectra are indicated with black arrows.

The smaller *k*_DTET_ value of the [Cr(bpmp)_2_]^3+^/DPA pair is more than compensated by the very long excited state lifetime *τ*_0_=890 μs of [Cr(bpmp)_2_]^3+^. The DTET efficiencies *Φ*_DTET_=1−*τ*/*τ*_0_ reach values of 84 % and >90 % for DPA concentrations of 100 μM and >300 μM (saturation), respectively. These values strongly exceed *Φ*_DTET_=69 % of the only other DTET process to an organic dye reported so far, that involved a luminescent doublet π‐radical sensitizer with *τ*_0_ of only 27 ns^[25]^ and DPA (5 mM), as well as *Φ*_TTET_=56 % of the classical [Ru(bpy)_3_]^2+^/DPA pair (*τ*_0_=935 ns, Figure 2b). This underlines the importance of long sensitizer excited state lifetimes for these processes.

Formation of the T_1_ state of DPA (^3^DPA) was assessed by nanosecond TA spectroscopy for higher concentrations of [Cr(bpmp)_2_]^3+^ and DPA than those used for the luminescence studies. This yields the characteristic and essentially solvent independent 445 nm ^3^DPA absorption band[^1l^, ^22^, ^26^] (associated with electronic transitions from the long‐lived lowest triplet state to higher triplet states of DPA) 10 μs after the excitation pulse (Figure 2, Figure S5; 2 mM [Cr(bpmp)_2_]^3+^/1 mM DPA) and confirms DTET from the excited Cr^III^ complex to DPA (Figure 2a, middle panel). The ISC process (Figure 1c) of the initially excited quartet state ^4^T_2_ to the doublet states ^2^E/^2^T_1_ and vibrational relaxation (VR, Figure 1c) are much faster according to reported fs transient absorption spectroscopic data of [Cr(bpmp)_2_]^3+^.^[14e]^ In the absence of DPA (Figure 2a, top panel), the TA spectrum of the relaxed doublet state is observed 100 ns after the excitation pulse. Due to the large energy gap between ^4^T_2_ and ^2^E/^2^T_1_ states, back‐ISC is thermodynamically unfeasible (Figure 1b). To gain a deeper insight into the formation efficiency of the DPA triplet and to quantify the involved processes, we performed quantitative TA studies with the [Cr(bpmp)_2_]^3+^/DPA pair utilizing [Ru(bpy)_3_]^2+^ as a reference sensitizer.^[27]^ As TTET from [Ru(bpy)_3_]^2+^ to anthracenes proceeds without side reactions as demonstrated by the characteristic ^3^DPA spectrum and the absence of DPA radical ion formation (indicated by the absence of additional absorption bands in the red spectral region in the lower panel of Figure 2a),[^1j^, ^1l^, ^28^] this system is a suitable actinometer in laser flash photolysis (LFP) studies.^[27]^ Thereby, the efficiency of the combined ISC and DTET processes of the [Cr(bpmp)_2_]^3+^ sensitizer was determined to *Φ*_ISC+DTET_=(92±5) % using a TTET efficiency of [Ru(bpy)_3_]^2+^ of 56 % (Figure 2b; Supporting Information, Figure S6).

As shown in Figure 2b, the ^3^DPA decay in the presence of [Cr(bpmp)_2_]^3+^ is faster than that observed for [Ru(bpy)_3_]^2+^.ek; This suggests a back‐energy transfer process, namely triplet‐doublet energy transfer (TDET) from ^3^DPA to [Cr(bpmp)_2_]^3+^ in its ^4^A_2_ ground state which is thermodynamically feasible (Figure 1c). TDET manifests itself also in the time‐resolved luminescence spectra of [Cr(bpmp)_2_]^3+^ in the presence of DPA recorded with delays of 20 ns and 50 μs, revealing prompt and delayed [Cr(bpmp)_2_]^3+^ phosphorescence, respectively (Figure S7).

Beyond the small enthalpy difference between the ^2^E/^2^T_1_ manifold of [Cr(bpmp)_2_]^3+^ and the T_1_ state of DPA (Figure 1c), entropy aspects can be relevant for this equilibrium.^[29]^ Assuming that i) only the lowest microstate of the ^2^E/^2^T_1_ manifold of [Cr(bpmp)_2_]^3+^ is significantly populated (a ^2^T_1_‐derived microstate; Figure 1d^[14e]^ and ii) the spin conservation rules^[30]^ do merely affect the kinetic feasibility and not the thermodynamics, only the spin degeneracies play a role (Figure 1d). The number of degenerate states of the [^4^A_2_(Cr)+T_1_(DPA)] combination (*n*=4×3=12) then exceeds the number of the [^2^T_1_(Cr)+S_0_(DPA)] combinations (*n*=2×1=2) (Figure 1c). This yields an upper limit for the entropic contribution of Δ*S*=R ln(12/2)=15 J mol^−1^ K^−1^, specific for Cr^III^ doublet sensitizer/triplet annihilator pairs and the DTET process. This adds an amount of ≈4.5 kJ mol^−1^ to the driving force Δ*G* of the forward DTET at room temperature. In contrast, no entropic effects are expected for TTET with ^3^MLCT sensitizers such as [Ru(bpy)_3_]^2+^. Even without such electronic entropy effects (i.e. for conventional TTET reactions), uphill energy transfer processes can occur at room temperature. This has been recently observed for an Ir^III 3^MLCT sensitizer and benzothiophenes (triplet energy difference of 24 kJ mol^−1^).^[31]^ The feasibility of this up‐hill process was attributed to the thermal population of vibrational or rotational levels of the excited sensitizer or ground‐state acceptor molecules which helped to overcome the thermodynamic energy gap.^[31]^

Consequently, [Cr(bpmp)_2_]^3+^ and DPA engage in an excited state equilibrium due to their long‐excited state lifetimes, similar excited state energies, and the available thermal energy to overcome small energy gaps. As the doublet states, which can be reached by direct excitation of [Cr(bpmp)_2_]^3+^ and by repopulation via TDET, form an excited‐state reservoir, no energy is lost. Hence, the TDET/DTET energy transfer pathways seem to barely affect the following TTA process (Figure 1c) and the overall UC process is very efficient despite the reduced lifetime of the ^3^DPA state (see below).

**Figure 3:**
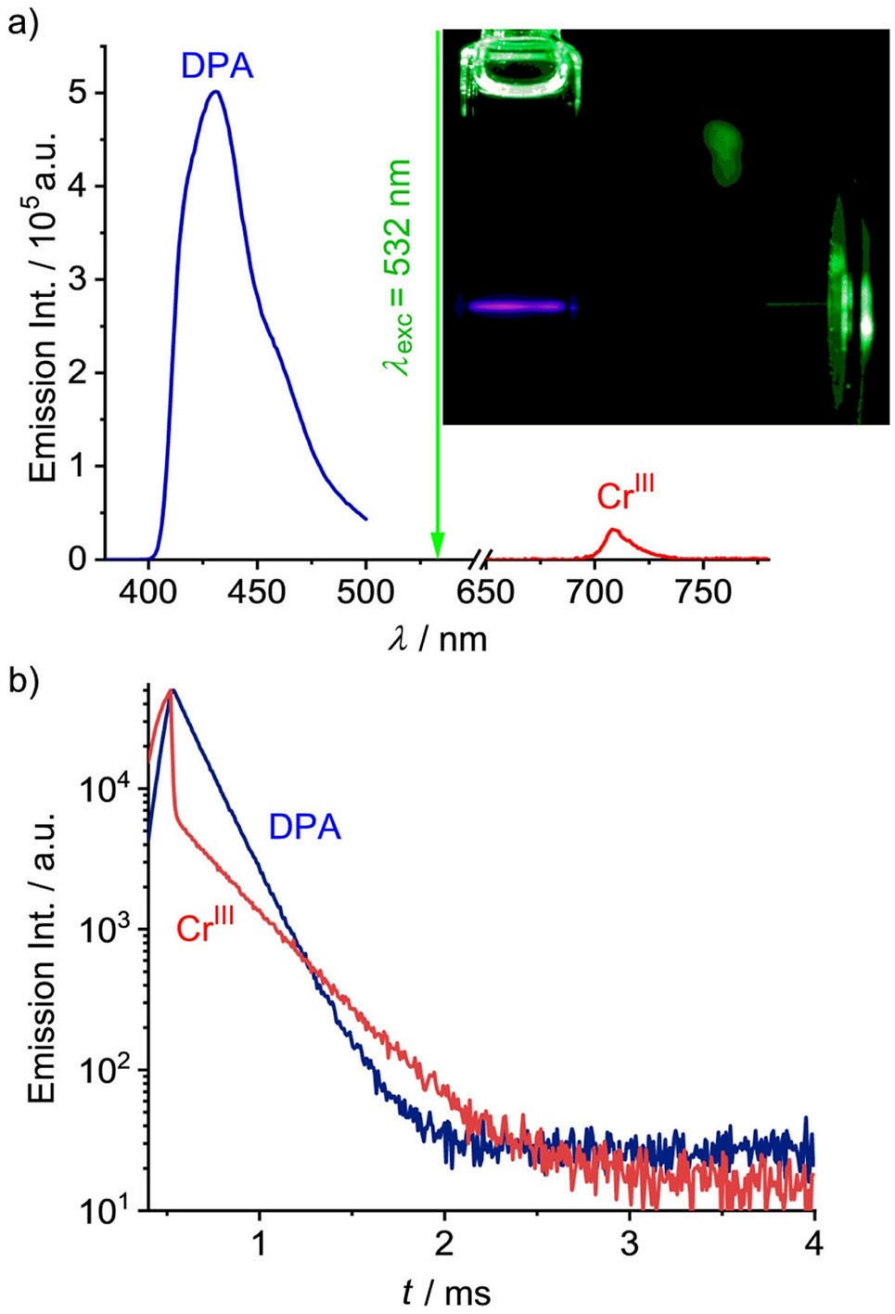
a) UCL spectrum (532 nm, cw, 1.5 W cm^−2^) and b) UCL decay (532 nm, 250 Hz, pulse width 500 μs) of [Cr(bpmp)_2_]^3+^/DPA (blue/red). The red emission trace in (b) corresponds to the concomitant [Cr(bpmp)_2_]^3+^ phosphorescence decay. Inset: Photograph of the sample under 532 nm laser excitation (laser power ≈40 mW). The sensitizer and acceptor concentrations in deoxygenated acidified DMF were 50 μM and 1 mM.

Excitation of an oxygen‐free acidified DMF solution of [Cr(bpmp)_2_]^3+^ and DPA with a 532 nm laser leads to strong blue DPA fluorescence at 432 nm (Figure 3a). The UCL maximum is blue shifted by 0.54 eV relative to the 532 nm excitation (Figure 1b, Figure 3a). In the absence of [Cr(bpmp)_2_]^3+^, no DPA fluorescence occurs under these conditions (Figure 1b; Figure S9). The integrated UCL intensity *I*_400–500_ exceeds the residual emission of [Cr(bpmp)_2_]^3+^ (*I*_680–750_) by a factor of about 45 indicating an efficient TTA‐UC process. Even excitation at 532 nm with a significantly less intense xenon lamp (≈1 mW cm^−2^) leads to noticeable UCL of DPA (Figure S10).

Increasing the DPA concentration enhances the UCL signal, which gradually approaches saturation (Figure S11). The fluorescence lifetime of directly excited DPA equals 6.6 ns, while the lifetime of the DPA fluorescence fed by green light excitation of [Cr(bpmp)_2_]^3+^ and DTET/TTA amounts to 162 μs (Figure 3b). The T_1_ state of DPA has a millisecond lifetime in the absence of non‐radiative processes.^[32]^ Consequently, the long UC fluorescence lifetime of 162 μs confirms its delayed nature caused by the intermediate population of the long‐lived T_1_ state of DPA (Figure 1c).

Time‐resolved phosphorescence measurements of solutions of the [Cr(bpmp)_2_]^3+^/DPA pair (50 μM/1 mM; *λ*_exc_=532 nm, *λ*_obs_=709 nm) reveal biexponential decay kinetics with lifetimes *τ*_1_=7 μs (15 %) and *τ*_2_=303 μs (85 %), assigned to the prompt and delayed phosphorescence of [Cr(bpmp)_2_]^3+^, respectively (Figure 3b). The delayed signal arises from the doublet states of [Cr(bpmp)_2_]^3+^ which can be repopulated by TDET from the long‐lived T_1_ state of DPA (Figure 1c, d). An increase in the sensitizer concentration leads to a reduction in the delayed phosphorescence lifetime and an increase in its contribution to the luminescence decay (enlarged relative amplitude, see Supporting Information for details, Figure S8, Table S1). These data agree well with the assumed excited state equilibrium of [Cr(bpmp)_2_]^3+^ and DPA involving DTET/TDET processes (Figures 1c, d).^[33]^ Under these conditions and using the lifetime of the prompt [Cr(bpmp)_2_]^3+^ phosphorescence *τ*_1_ of 7 μs, the DTET yield amounts to *Φ*_DTET_=1−*τ*_1_/*τ*_0_=99.2 % in agreement with the high DPA triplet quantum yield determined by quantitative TA spectroscopy. Notably, these values imply a lower limit for the ISC efficiency of [Cr(bpmp)_2_]^3+^ on the order of 90 %. This high value is remarkable considering that chromium is a first‐row transition metal with a lower spin‐orbit coupling constant compared to precious metals such as ruthenium.^[34]^

**Figure 4:**
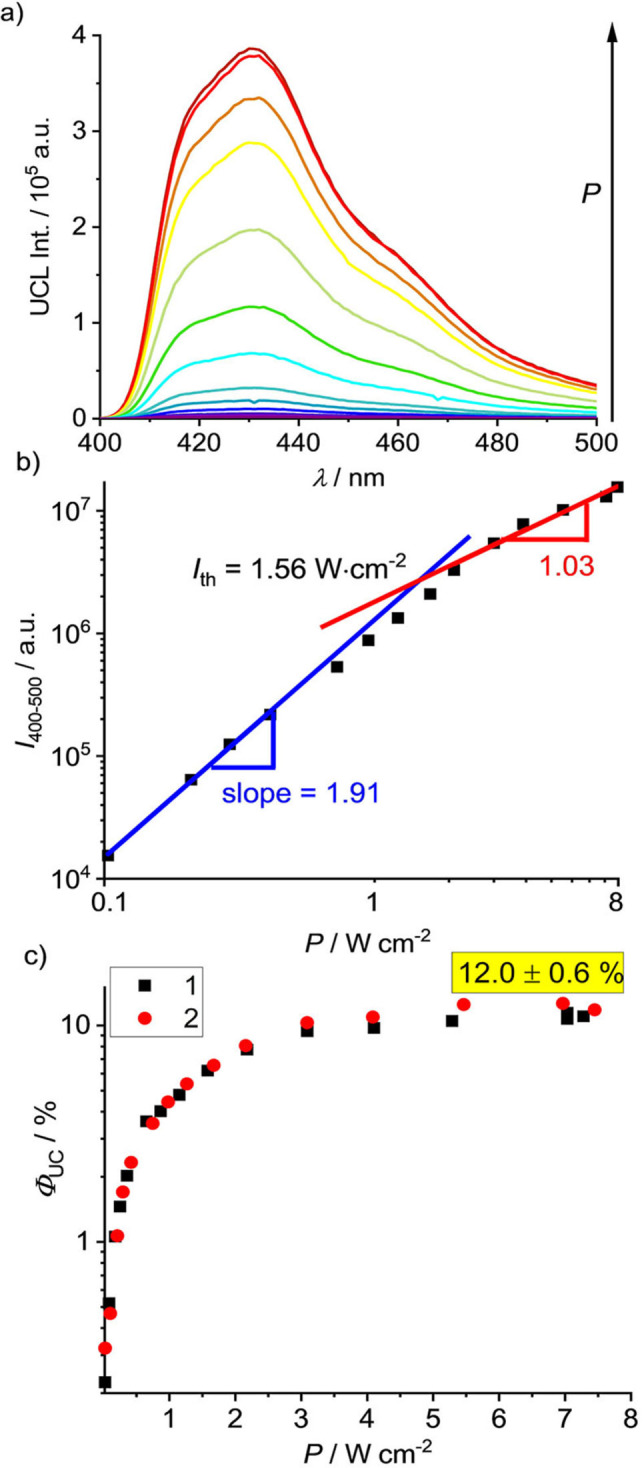
a) UCL spectra of [Cr(bpmp)_2_]^3+^/DPA (50 μM/1 mM in deoxygenated acidified DMF) as a function of excitation power density of a 520 nm laser (cw, ≈8 W cm^−2^), b) excitation power density dependence of the integrated UCL (*I*_400–500_) of DPA, c) relatively determined *Φ*_UC_ of the [Cr(bpmp)_2_]^3+^/DPA pair as a function of excitation power density of the 520 nm laser; the red and black symbols denote two independent experiments.

Due to the biphotonic nature of sTTA‐UC, UCL from sensitizer/annihilator pairs nonlinearly depends on excitation power density and shows a slope factor (photonic order) of about two in the non‐saturated regime which approaches one upon saturation. Increasing the excitation power density of the 532 nm laser (cw, 1.5 W cm^−2^) drastically enhances the UCL of DPA (Figure 4a). The integrated UCL (*I*_400–500_) of the [Cr(bpmp)_2_]^3+^/DPA pair shows a slope factor of 1.88 as expected for a biphotonic process. Yet, saturation is not reached up to power densities of 1.5 W cm^−2^ (Figure S12). To determine *Φ*_UC_ for the saturated system, we utilized a more intense 520 nm laser (cw, ca. 8 W cm^−2^). Under these conditions, the integrated UCL nearly quadratically (slope factor of 1.91) depends on power density for power densities of up to about 2 W cm^−2^ and then starts to saturate as indicated by the linear slope of 1.03. The power density threshold value *I*_th_, which indicates the turning point from a biphotonic to a linear (monophotonic) process reflecting UC saturation, amounts to about 1.56 W cm^−2^ (Figure 4b). This value, which is relatively high compared to *I*_th_ values of sTTA‐UC systems reported for systems utilizing Zn^II^, Zr^IV^, and Cu^I^ sensitizers,[^6d^, ^11d^, ^35^] is ascribed to the very small absorption of [Cr(bpmp)_2_]^3+^ above 520 nm (*ϵ*≈30–45 M^−1^ cm^−1^).^[14e]^ This leaves room for improvement of the sTTA‐UC by further red shifting the absorption band the of the Cr^III^ sensitizer and/or increasing its molar absorption coefficient for green light excitation. The relative determination of *Φ*_UC_ (50 μM [Cr(bpmp)_2_]^3+^/1 mM DPA) give *Φ*_UC_=8.7 % for excitation with the 532 nm laser and *Φ*_UC_=(12.0±0.6) % for 520 nm excitation under saturated conditions (Figure 4c, Figure S12, S13). This value is amongst the highest UC efficiencies reported so far for sensitizers containing only earth‐abundant elements.[^1l^, ^6d^, ^11d^, ^35a^]

With *Φ*_F_ of DPA of 82.8 % (measured in diluted, acidified DMF solution), *Φ*_DTET+ISC_ of about 90 % and a spin‐statistical factor for DPA of *f*=40 %, Equation (1) gives an upper limit of the sTTA‐UC quantum yield *Φ*_UC_ of about 15 %. This value is close to the actual *Φ*_UC_=(12.0±0.6) % achieved with [Cr(bpmp)_2_]^3+^/DPA. The slight discrepancy is probably due to optical losses^[2b]^ resulting from the higher DPA concentration. In addition to this relatively high sTTA‐UC efficiency, the excellent photostability of [Cr(bpmp)_2_]^3+^ and DPA enable a constant UCL intensity under continuous laser illumination at 532 nm for more than two hours (Figure S14).

Sterically less‐hindered anthracene derivatives form [4+4] cycloaddition products under UV illumination by the reaction of an anthracene in its S_1_ state with a ground state anthracene.^[36]^ Alternative to direct excitation with UV light, the S_1_ state of these anthracenes can also be populated via sTTA‐UC, e.g., by using [Ru(bpy)_3_]^2+^ derivatives as sensitizer and excitation with a 457.9 nm laser, yielding the respective anthracene dimer.^[1i]^ To demonstrate that the [Cr(bpmp)_2_]^3+^ sensitizer can also initiate photochemical reactions via sTTA‐UC, anthracene‐9‐propionic acid (APA), 9‐anthracene carboxylic acid (ACA), and anthracene (An) were illuminated with 532 nm light in the presence of [Cr(bpmp)_2_]^3+^ in acidified deoxygenated DMF. All annihilators quench the [Cr(bpmp)_2_]^3+^ emission as expected from their T_1_ energies, which are similar to that of DPA (Figure S15). UCL is observed in the steady‐state and time‐resolved spectra, showing the characteristic UC dependence on *P* (Figures S16–S21). Yet, the *Φ*_UC_ values obtained for APA, ACA, and An are much lower than *Φ*_UC_ of [Cr(bpmp)_2_]^3+^/DPA (Figure S22, Table S2). Photolysis at 532 nm in the presence of [Cr(bpmp)_2_]^3+^ leads to a decrease of the absorption bands and UCL of the anthracenes (Figures S23, S24). Exemplary for the [Cr(bpmp)_2_]^3+^/An pair, anthracene photodimerization was confirmed by proton NMR spectroscopy. The ^1^H NMR spectra of [Cr(bpmp)_2_]^3+^/An in oxygen‐free acidified d_7_‐DMF obtained during photolysis with a 525 nm Kessil LED indicated the formation of the [4+4] dimer on the basis of the increasing characteristic ^1^H NMR resonance at *δ*=4.47 ppm for the bridgehead protons of the anthracene dimer (Figure S25).^[1i]^

## Conclusion

The Cr^III^ complex [Cr(bpmp)_2_]^3+^ can replace classical ^3^MLCT sensitizers, in particular [Ru(bpy)_3_]^2+^ derivatives, in sensitized triplet‐triplet annihilation upconconversion (sTTA‐UC) processes with 9,10‐diphenyl anthracene (DPA) as the annihilator generating upconverted blue photons from green photons. The key to success for efficient sTTA‐UC with [Cr(bpmp)_2_]^3+^ lies in its comparably low excitation energy (2.33 eV), its relatively high doublet state energy (1.75 eV), its high photostability, and especially in its long doublet state lifetime of 890 μs. The latter can overcompensate the comparably lower doublet‐triplet energy transfer rate, leading to high energy transfer efficiencies close to unity, thereby even outperforming [Ru(bpy)_3_]^2+^. This high energy transfer efficiency furnishes a UC quantum yield *Φ*_UC_ of (12.0±0.6) %. For the sterically less‐hindered anthracene derivatives anthracene‐9‐propionic acid, 9‐anthracene carboxylic acid, and anthracene, the [Cr(bpmp)_2_]^3+^ sensitizer enables a [4+4] cycloaddition with green light that traditionally requires UV light.

Overall, this proof‐of‐concept study paves the way for novel sTTA‐UC photosensitizers based on earth‐abundant metal ions using long‐lived spin‐flip excited states instead of the traditionally employed precious metal sensitizers relying on charge‐transfer excited states. To further lower the excitation power density threshold of sTTA‐UC, on‐going studies aim for increasing the absorptivity of the Cr^III^ sensitizers in the visible region.

## Conflict of interest

The authors declare no conflict of interest.

1

## Supporting information

As a service to our authors and readers, this journal provides supporting information supplied by the authors. Such materials are peer reviewed and may be re‐organized for online delivery, but are not copy‐edited or typeset. Technical support issues arising from supporting information (other than missing files) should be addressed to the authors.

Supporting Information

## Data Availability

All experimental data, procedures for data analysis and pertinent data sets are provided in the Sup. Inform. document. Further raw data sets are available from the authors upon reasonable request.
