# Supplementary material for: Efficient Triplet‐Triplet Annihilation Upconversion Sensitized by a Chromium(III) Complex via an Underexplored Energy Transfer Mechanism
Source: Angew Chem Int Ed Engl. 2022 May 9;61(27):e202202238. doi: 10.1002/anie.202202238 (PMC9322448; doi:10.1002/anie.202202238)
Supplement: Supplementary file 1 — Supporting Information [file ANIE-61-0-s001.pdf]

Angewandte Chemie
Eine Zeitschrift der Gesellschaft Deutscher Chemiker

Supporting Information

Efficient Triplet-Triplet Annihilation Upconversion Sensitized by a Chromium(III) Complex via an Underexplored Energy Transfer Mechanism

C. Wang, F. Reichenauer, W. R. Kitzmann, C. Kerzig, K. Heinze*, U. Resch-Genger**

Supporting Information

Content

1. Materials and methods 2

2. Optical characterization of the sensitizer and the annihilators 4

3. Stern-Volmer studies 5

4. Density functional theory calculations 6

5. Transient absorption measurements of ^{3}DPA 10

6. Delayed sensitizer phosphorescence measurements 11

7. Photon upconversion studies 12

8. Photon upconversion and photodimerization with other anthracenes 15

9. References 27

1. Materials and methods

$[Cr(bpmp)_2][PF_6]_3$ was synthesized according to a reported procedure.[1] The anthracene derivatives were commercially obtained: 9,10-diphenylanthrace (DPA, Thermo Fisher Scientific), anthracene-9-propionic acid (APA, abcr), 9-anthracene carboxylic acid (ACA, Sigma-Aldrich), and anthracene (An, Sigma-Aldrich or Acros Organics). *N*,*N*-Dimethylformamide (DMF, anhydrous, 99.8%**,** Sigma-Aldrich), acetonitrile (ACN, HPLC grad**,** Sigma-Aldrich) and D_7-DMF (99.5 % D, Deutero), perchloric acid (70 % aq. soln., Alfa Aesar or Bernd Kraft) and $[Ru(bpy)_3][PF_6]_2$ (Sigma-Aldrich) were used as received from commercial suppliers.
To exclude the oxygen influence, all sample solutions for spectroscopic measurements were purged with argon for ca. 30 min in a sealed long-neck quartz cuvette.

UV/Vis absorption spectra were recorded with the Varian Cary 5000 spectrometer using 1.0 cm cells. Particularly, as the absorbance of the low-concentrated chromium complex is very low, absorption spectra for the Φ_{UC} determination were collected with very low measuring speed (0.1 nm/0.2 s) to suppress the influence from the noise and background.

Luminescence measurements (direct excitation): Phosphorescence spectra and decays of the $[Cr(bpmp)_2][PF_6]_3$ (sensitizer) were obtained with a calibrated spectrofluorometer FSP 920 from Edinburgh Instruments. For the measurement of the emission spectra, a CW xenon lamp was used as the excitation light source, while the time-resolved luminescence measurements were performed with a µs xenon flashlamp (100 Hz) and single photon counting detection in a multi-channel scaling mode. Fluorescence spectra and decays of the anthracene derivatives (acceptors) were measured on a calibrated spectrofluorometer FLS 920 from Edinburgh Instruments. The emission spectra were obtained with a CW xenon lamp as the excitation light source, while the decay kinetics were measured under direct excitation of DPA (395 nm), APA (375 nm), ACA (375 nm), and An (368 nm) with a supercontinuum laser (NKT FIU 15) (9.7 MHz) as the light source and a microchannel plate photomultiplier tube (MCP-PMT; R3809U50) (Figure S1).

UC luminescence measurements: UCL emission spectra were obtained with the calibrated spectrofluorometer FLS 920 equipped with a 532 nm CW laser (62 mW, Sunshine electronics, Shenzhen, CN) and a 520 nm CW laser (800 mW, Roithner Lasertechnik GmbH, Austria). One 532 nm notch filter was placed between the sample holder and the detector to suppress the excitation signal observed on the detector for the UC measurements using the 532 nm laser, while a metal filter (3926B, 9.12% transmission) was set between the sample and detector to reduce the too strong UC signal excited by the 520 nm laser. To take into account the various responses of the detector in different spectral regions, the emission of the UC signal (380-500 nm) and of the sensitizer (650-800 nm) were obtained with the corrective curve under strictly identical conditions (exc. and em. slit of 6 nm, using polarizers in the excitation and emission channel set to 0° and 54.7°, respectively). For excitation power density (*P*) dependent UCL emission measurements, the laser power was varied with a tunable OD filter and determined with a power meter (Newport 841-PE Powermeter) during the measurements. The laser spot size at the sample position was determined to be ca. 4 mm^2 (532 nm laser, basically identical spot sizes at different powers) and 5-8 mm^2 (520 nm laser, spot size differs a bit at different power) with a laser beam profiler (Newport, LBP2-HR-VIS). To exclude the different size effect on the calculated power density, the 520 nm-laser spot size was measured at each used laser power ranging from 0.3 mW to ca. 590 mW. The laser power density was calculated by dividing the measured laser power by the determined spot size.

UC luminescence decays of the annihilators were obtained on a calibrated spectrofluorometer FS5 from Edinburgh Instruments equipped with the same 532 nm laser (62 mW) and single photon counting detection in a multi-channel scaling mode. The laser was modulated in a pulsed mode (250 Hz, pulse width 500 µs) by a function generator (SRS, model DS345).

***Φ_{PL}* and *Φ_F* measurements:** The photoluminescence quantum yields of the sensitizer (50 µM, in deaerated DMF containing 0.1 M $HClO_4$) and the annihilators (10-15 µM, in air-saturated DMF containing 0.1 M $HClO_4$) were determined absolutely using a calibrated Ulbricht integrating sphere setup (Quantaurus-QY C11347-11, Hamamatsu). These measurements were carried out by direct excitation of $[Cr(bpmp)_2][PF_6]_3$ (462 nm), DPA (395 nm), APA (375 nm), ACA (375 nm), and An (368 nm), respectively, with the excitation wavelengths being given in brackets. For DPA, fluorescence quantum yield measurements in air-saturated and deoxygenated acidified DMF revealed only a very small influence of oxygen under these conditions that was not further quantified. The relative uncertainties of these measurements are estimated to be ±5 %.[2]

***Φ_{UC}* determination:** Φ_{UC} of UC samples containing 50 µM $[Cr(bpmp)_2][PF_6]_3$ and 1 mM anthracenes was determined relatively using a $[Cr(bpmp)_2][PF_6]_3$ (50 µM) solution without annihilator as reference.[3] Φ_{UC} was calculated according to Eq. S1.[4] In Eq. S1, A_{Ref} and A_{UC} stand for the absorbances of the reference solution and the UC sample at the excitation wavelength. The absorbance readouts at 532 and 520 nm were averaged by taking values from 533 to 531 nm and from 517 to 520 nm, respectively. I_{UC} and I_{Ref} represent the integrated intensities of the UCL and the reference emission, respectively. Φ_{Ref}, which equals the phosphorescence quantum yield of $[Cr(bpmp)_2][PF_6]_3$ in deaerated acidified DMF solution at room temperature, is 9.2%. Both reference and UC samples were prepared and measured twice and independently on different days.

$$\Phi_{\mathrm{UC}} = \Phi_{\mathrm{Ref}} \times \frac{A_{\mathrm{Ref}}}{A_{\mathrm{UC}}} \times \frac{I_{\mathrm{UC}}}{I_{\mathrm{Ref}}} \qquad \text{(Eq. S1)}$$

To confirm the linear power density dependence of the $[Cr(bpmp)_2][PF_6]_3$ phosphorescence, the corrected emission spectra of the reference solution were measured as a function of increasing power densities of the 520 nm and the 532 nm laser used as excitation light sources, respectively (Figure S13).

UC luminescence decays of the annihilators were obtained on a calibrated spectrofluorometer FS5 from Edinburgh Instruments equipped with the same 532 nm laser (62 mW) and single photon counting detection in a multi-channel scaling mode. The laser was modulated in a pulsed mode (250 Hz, pulse width 500 µs) by a function generator (SRS, model DS345).

Stern-Volmer studies with DPA and other anthracenes were performed by measuring the phosphorescence intensity (I_{709}) and lifetime (τ_{709}) of the deaerated $[Cr(bpmp)_2][PF_6]_3$ solutions (0.5 mM in acidified DMF) in the presence of the anthracene annihilators of different concentrations (0 – 1 mM), respectively.

Laser flash photolysis (LFP) experiments were carried out with an LP980KS apparatus from Edinburgh Instruments, monitoring either transient absorption or emission signals. A frequency-doubled Nd:YAG laser from Litron (Nano LG 300-10) was used for selective excitation of the sensitizers with laser pulses of ~5 ns duration. The excitation intensity at 532 nm was adjusted by a step-motor driven attenuator. Typical laser pulse energies were 40 mJ and the laser output stability was confirmed by several control experiments during each series of experiments. Detection of transient absorption and time-gated emission spectra occurred on an iCCD camera from Andor with precisely adjustable detection delay time (relative to the laser pulse) and integration time. Kinetic

traces at selected wavelengths were recorded using a photomultiplier tube. All LFP experiments were performed at 293 K with a cuvette holder allowing temperature control. The DMF solutions for these experiments were prepared in long neck cuvettes with an air-tight teflon screwcap in an Ar-filled glove box (MBraun Unilab Eco, <5 ppm oxygen).

NMR spectra were recorded on a Bruker Avance DRX 400 spectrometer at 400.31 MHz (^{1}H). Resonances are reported in ppm versus the solvent signal as internal standard.

Photolysis of anthracene in the presence of [Cr(bpmp)$_2$][PF$_6$]$_3$. A 0.1 M solution of D$_7$-DMF (99.5 % D, Deutero) and perchloric acid (70 % aq. soln.) was de-oxygenated by six freeze-pump-thaw cycles under pressures of less than 5×10^{-2} mbar. A 0.1 M solution of anthracene and 0.005 M solution of [Cr(bpmp)$_2$][PF$_6$]$_3$ in this solvent mixture was irradiated in an NMR tube with a Kessil LED (Kessil Science, PR160L, 525 nm (max. 44 W), 100-240 VAC, 352 mW cm^{-2} measured at 1 cm distance; www.kessil.com). ^{1}H NMR spectra were recorded after 0 min, 5 min, 15 min, 80 min, 5 h and 8 h of irradiation.

2. Optical characterization of the sensitizer and the annihilators

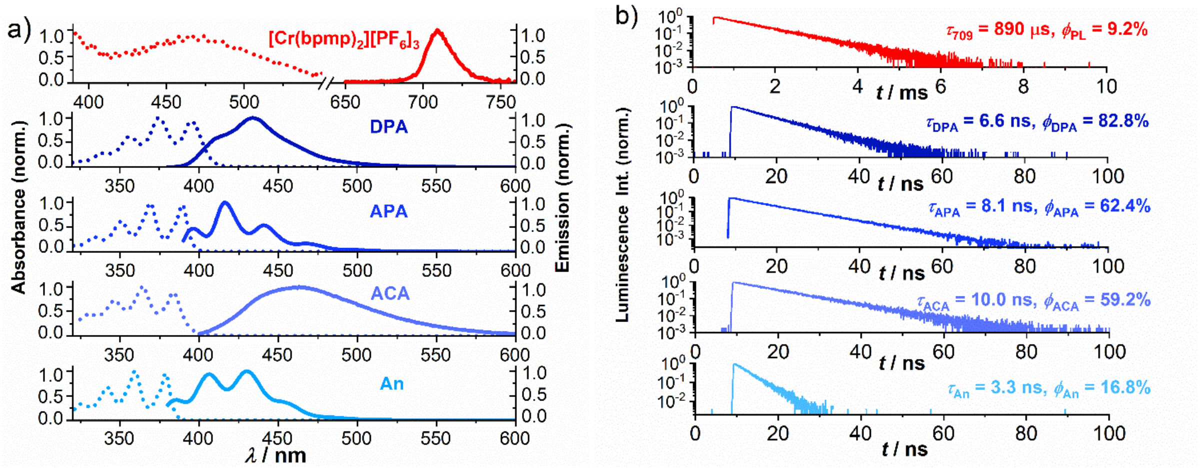

Figure S1: a) Absorption (dotted lines) and emission spectra (solid lines) of [Cr(bpmp)$_2$][PF$_6$]$_3$ (50 µM) and the annihilators DPA, APA, ACA, and An in acidified DMF (10 – 15 µM) at room temperature; The broad red-shifted emission spectrum of ACA observed in this solvent is ascribed to the formation of a linear-type ACA dimer bridged by hydrogen-bonds involving the COOH groups;[5] b) corresponding luminescence decays and quantum yields obtained for direct excitation at 462 nm, 395 nm, 375 nm, 375 nm, and 368 nm, respectively.

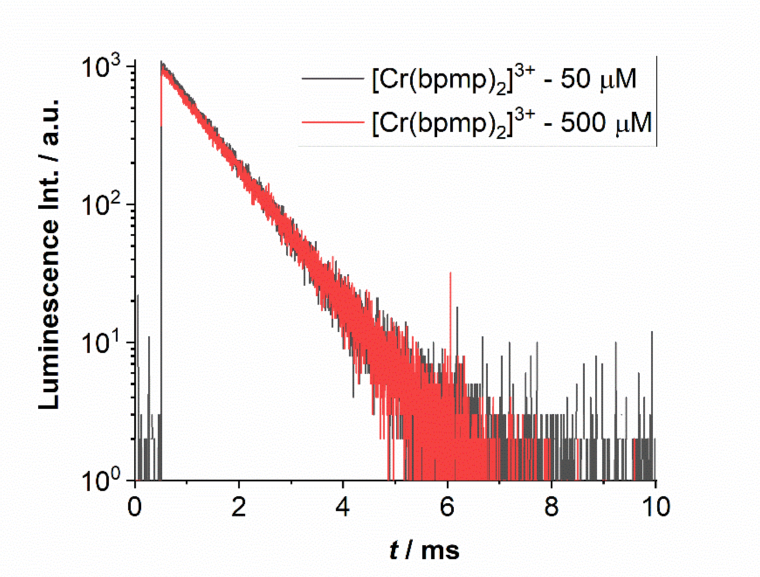

Figure S2: Phosphorescence decay kinetics of $[Cr(bpmp)_2][PF_6]_3$ at 709 nm in deaerated acidified DMF solution at room temperature obtained with concentrations of 50 µM (black) and 500 µM (red) of $[Cr(bpmp)_2][PF_6]_3$. Excitation was set at 532 nm with a xenon flash lamp.

3. Stern-Volmer studies

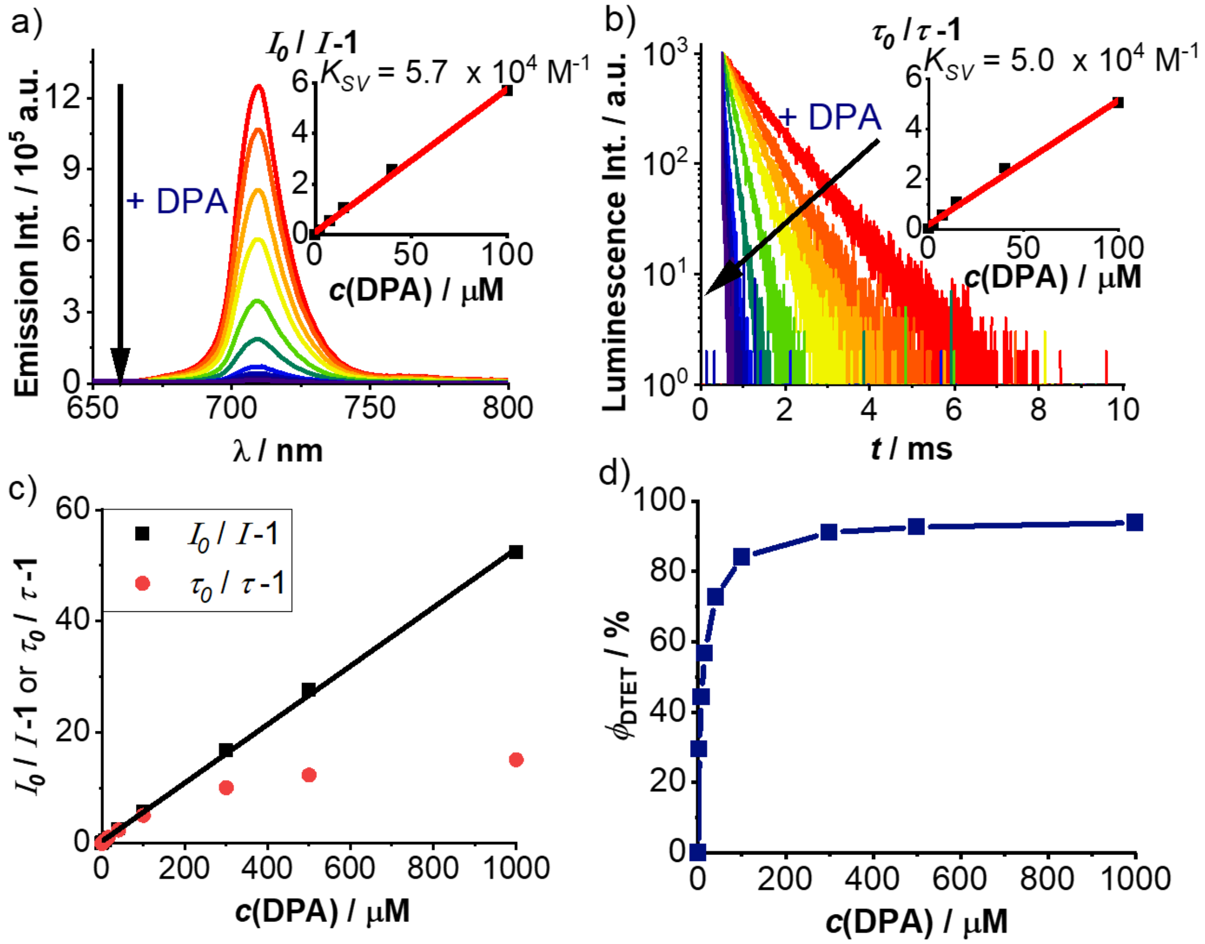

Figure S3: Stern-Volmer (SV) studies of $[Cr(bpmp)_2][PF_6]_3$ (0.5 mM) in acidified DMF in the presence of increasing concentrations of DPA: a) Phosphorescence intensity and b) Decay kinetics of $[Cr(bpmp)_2][PF_6]_3$ as a function of DPA concentration ((exc. 532 nm, em. 709 nm). The resulting SV plots up to a DPA concentration of 100 µM are given in the insets. c) SV plot derived from measurements of the phosphorescence intensity (dotted lines) and lifetime (solid lines) of $[Cr(bpmp)_2][PF_6]_3$ in the presence of increasing concentrations of DPA up to 1 mM; d) Φ_{DTET} derived from the sensitizer lifetimes plotted as a function of the DPA concentration.

Possible explanations for the deviations between the lifetime- and intensity-based SV plots could be static energy transfer and/or aggregation induced emission quenching, which does not yield the desired photoproduct. To address the observed deviations between lifetime- and intensity-based SV plots, transient absorption spectroscopic measurements (Figure S5 – S7) and luminescence lifetime measurements of the delayed phosphorescence of the sensitizer (Figure S8, Table S1) were performed. These data confirm the purely dynamic nature of the DTET process for the $[Cr(bpmp)_2][PF_6]_3$/DPA pair. More details follow in Sections 5 and 6.

4. Density functional theory calculations

All calculations were performed using the quantum computing suite ORCA 4.2.1.[6] Geometry optimization with DFT-methodology was performed using unrestricted Kohn-Sham orbitals DFT (UKS), the restricted Kohn-Sham (RKS) orbitals (for the anthracene derivatives in S_0 state), and the B3LYP functional[7] in combination with Ahlrichs' split-valence triple-ζ basis set def2-TZVPP for all atoms.[8] Tight convergence criteria were chosen for DFT-(U)KS calculations (keywords tightscf and tightopt). All DFT-(U)KS calculations make use of the resolution of identity (Split-RI-J) approach for the Coulomb term in combination with the chain-of-spheres approximation for the exchange term (COSX).[9] The zero order relativistic approximation was used to describe relativistic effects in all calculations (keyword ZORA).[10] Grimme's empirical dispersion correction D3(BJ) was employed (keyword D3BJ).[11] To account for solvent effects, a conductor-like screening model (keyword CPCM (DMF)) modeling DMF was used in all calculations.[12] Explicit counter ions and/or solvent molecules were neglected.

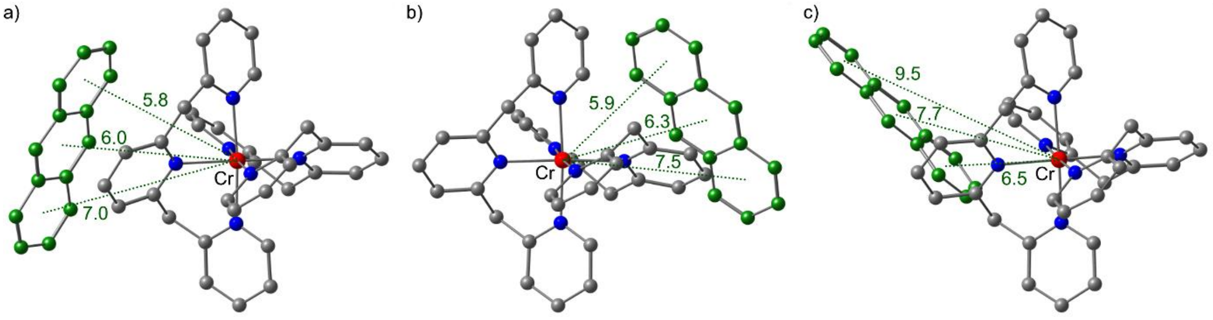

Figure S4: Geometries of $^4[[Cr(bpmp)_2]^{3+}$ + An] in three different arrangements optimized at the CPCM(DMF)-RI-B3LYP-D3BJ-ZORA/def2-TZVPP basis of theory. The anthracene acceptor is marked in green. The distances from Cr to the centers of the anthracene are given in Å. Hydrogen atoms are omitted for clarity.

Coordinates of DFT calculated geometries

^{4}[[Cr(bpmp)$_2$]$^{3+}$ + An]; orientation a); CPCM(DMF)-RI-B3LYP-D3BJ-ZORA/def2-TZVPP

24	-0.551561000	-0.171258000	0.523864000
7	-0.875026000	0.047765000	-1.558937000
7	-2.515539000	0.424947000	1.016303000
7	0.023703000	1.854078000	0.716366000
7	1.449136000	-0.594084000	0.002525000
7	-0.325913000	-0.541436000	2.586221000
6	1.697056000	-1.510733000	-0.955299000
6	2.483402000	0.033479000	0.596178000
6	2.972760000	-1.858886000	-1.333496000
6	3.794143000	-0.274301000	0.256000000
6	4.046974000	-1.235245000	-0.709662000
7	-1.007998000	-2.223221000	0.306823000
6	1.260030000	2.164599000	1.177448000
6	-0.811644000	2.847572000	0.328147000
6	1.690482000	3.479107000	1.241550000
6	-0.421594000	4.175637000	0.386800000
6	0.844238000	4.498822000	0.842077000
6	-3.013565000	1.621541000	0.652605000
6	-3.265594000	-0.393348000	1.783432000
6	-4.285162000	2.014225000	1.050914000
6	-4.530517000	-0.059548000	2.207709000
6	-5.055078000	1.172281000	1.834052000
6	-0.252770000	-3.143840000	0.949674000
6	-1.794404000	-2.614953000	-0.728204000
6	-0.208638000	-4.459660000	0.518073000
6	-1.756295000	-3.909159000	-1.207758000
6	-0.934678000	-4.840618000	-0.594767000
6	-1.887216000	-0.591122000	-2.180912000
6	-0.088882000	0.882346000	-2.270375000
6	-2.168533000	-0.351259000	-3.517794000
6	-0.310447000	1.149838000	-3.602976000
6	-1.382966000	0.534232000	-4.237867000
6	0.118934000	-1.722196000	3.058006000
6	-0.729374000	0.406222000	3.460506000
6	-0.705518000	0.216576000	4.823384000
6	0.160504000	-1.971529000	4.423865000
6	-0.249949000	-0.999188000	5.318694000
6	0.628790000	-2.757229000	2.105054000
6	-2.677434000	-1.599645000	-1.396352000
6	-2.181567000	2.525624000	-0.206014000
6	2.187363000	1.072427000	1.638025000
9	-2.189427000	-6.088050000	2.716619000
9	-3.129233000	-5.594464000	1.766322000
9	-3.422979000	-6.284233000	0.590848000
9	-3.749892000	-4.317925000	2.006276000
9	-4.599260000	-3.782574000	1.037950000
9	-4.850772000	-4.448971000	-0.162056000
9	-4.262687000	-5.743101000	-0.383763000
9	-1.892156000	-5.377059000	3.842860000
9	-3.451429000	-3.635100000	3.220601000
9	-5.639179000	-3.867208000	-1.195551000
9	-4.504496000	-6.398818000	-1.625284000
9	-5.261019000	-5.804856000	-2.593797000
9	-5.829290000	-4.518875000	-2.379359000
9	-2.543345000	-4.140611000	4.104715000

^{4}[[Cr(bpmp)$_2$]$^{3+}$ + An]; orientation b); CPCM(DMF)-RI-B3LYP-D3BJ-ZORA/def2-TZVPP

24	-0.236839000	-0.226109000	-0.207425000
7	0.128359000	1.367329000	-1.539357000
7	-2.261617000	0.296555000	0.084963000
7	0.230958000	0.965543000	1.485125000
7	1.838770000	-0.610360000	-0.390528000
7	-0.650020000	-1.911129000	0.977363000
6	2.396461000	-0.666181000	-1.617908000
6	2.629700000	-0.728878000	0.694160000
6	3.746930000	-0.847397000	-1.811005000
6	4.000634000	-0.903945000	0.563249000
6	4.569482000	-0.966302000	-0.697474000
7	-0.605727000	-1.448113000	-1.885214000
6	1.235802000	0.600201000	2.317898000
6	-0.456937000	2.105481000	1.736538000
6	1.580757000	1.374280000	3.412700000
6	-0.149276000	2.904427000	2.825814000
6	0.884561000	2.542157000	3.672153000
6	-2.617903000	1.511102000	0.547457000
6	-3.220635000	-0.624917000	-0.148727000
6	-3.951412000	1.826027000	0.773336000
6	-4.556584000	-0.366952000	0.053087000
6	-4.933282000	0.884525000	0.522892000
6	-0.133866000	-2.718149000	-1.915036000
6	-1.152250000	-0.914101000	-3.005867000
6	-0.141347000	-3.452353000	-3.089177000
6	-1.224931000	-1.636764000	-4.183978000
6	-0.697232000	-2.914555000	-4.235327000
6	-0.572153000	1.506304000	-2.683012000
6	1.113074000	2.247267000	-1.258475000
6	-0.296034000	2.541005000	-3.567243000
6	1.427525000	3.296202000	-2.092210000
6	0.707593000	3.449368000	-3.271408000
6	-0.264312000	-3.149423000	0.612351000
6	-1.294053000	-1.737613000	2.151774000
6	-1.557254000	-2.778040000	3.012778000
6	-0.491764000	-4.234647000	1.448410000
6	-1.137631000	-4.054297000	2.658640000
6	0.461411000	-3.352303000	-0.684899000
6	-1.651145000	0.504147000	-2.986192000
6	-1.556768000	2.538421000	0.806370000
6	2.002736000	-0.665871000	2.054913000
9	-3.703305000	-4.729060000	-0.106477000
9	-3.119588000	-5.149691000	-1.336580000
9	-1.991380000	-5.969208000	-1.383341000
9	-3.733295000	-4.729731000	-2.569298000
9	-3.193520000	-5.161588000	-3.779953000
9	-2.067518000	-5.984321000	-3.825699000
9	-1.445663000	-6.394035000	-2.594079000
9	-4.828441000	-3.957941000	-0.093020000
9	-4.893038000	-3.902244000	-2.510159000
9	-1.481134000	-6.397204000	-5.056188000
9	-0.262113000	-7.186509000	-2.653392000
9	0.281104000	-7.540742000	-3.853937000
9	-0.338767000	-7.143995000	-5.071275000
9	-5.431978000	-3.538915000	-1.311006000

^{4}[[Cr(bpmp)$_2$]$^{3+}$ + An]; orientation c); CPCM(DMF)-RI-B3LYP-D3BJ-ZORA/def2-TZVPP

24	-0.063554000	-0.281434000	0.184843000
7	0.549493000	1.085623000	-1.320630000
7	-2.153985000	-0.060594000	-0.079397000
7	-0.125134000	1.268388000	1.607037000
7	2.002650000	-0.459480000	0.542986000
7	-0.643020000	-1.713381000	1.631621000
6	2.829754000	-0.724042000	-0.490657000
6	2.529525000	-0.226681000	1.761427000
6	4.198569000	-0.771044000	-0.353156000
6	3.903580000	-0.258556000	1.960771000
6	4.749816000	-0.533712000	0.900514000
7	-0.005056000	-1.826650000	-1.254738000
6	0.684125000	1.234408000	2.693121000
6	-0.972056000	2.313506000	1.439406000
6	0.671728000	2.257128000	3.627654000
6	-1.015519000	3.353400000	2.352099000
6	-0.184337000	3.330529000	3.458516000
6	-2.751967000	1.135169000	0.101876000
6	-2.920978000	-1.161920000	-0.221559000
6	-4.134243000	1.237593000	0.171841000
6	-4.296081000	-1.122742000	-0.158881000
6	-4.917748000	0.100860000	0.051406000
6	0.374765000	-3.078752000	-0.907098000
6	-0.337911000	-1.566503000	-2.541956000
6	0.415171000	-4.101695000	-1.841131000
6	-0.283416000	-2.551707000	-3.512232000
6	0.090643000	-3.837948000	-3.159741000
6	0.203843000	0.892706000	-2.607893000
6	1.423610000	2.068216000	-1.017962000
6	0.743450000	1.669582000	-3.622521000
6	1.985208000	2.880010000	-1.979158000
6	1.647577000	2.671641000	-3.310616000
6	-0.249475000	-2.998571000	1.541865000
6	-1.542970000	-1.366922000	2.576939000
6	-2.085341000	-2.279063000	3.453792000
6	-0.772358000	-3.966827000	2.388387000
6	-1.701354000	-3.610605000	3.352074000
6	0.777415000	-3.366528000	0.512367000
6	-0.790807000	-0.183723000	-2.916975000
6	-1.881207000	2.349946000	0.239919000
6	1.607296000	0.066196000	2.906588000
9	-4.185299000	-1.280890000	-3.530644000
9	-5.317925000	-2.131050000	-3.365282000
9	-5.214185000	-3.522775000	-3.371184000
9	-6.613107000	-1.523883000	-3.200599000
9	-7.738347000	-2.339938000	-3.086407000
9	-7.638237000	-3.731628000	-3.118033000
9	-6.339738000	-4.340353000	-3.250200000
9	-4.319151000	0.076566000	-3.522287000
9	-6.704224000	-0.101025000	-3.175382000
9	-8.785047000	-4.574382000	-3.032993000
9	-6.253464000	-5.764267000	-3.269341000
9	-7.376822000	-6.535222000	-3.182805000
9	-8.660212000	-5.932652000	-3.067700000
9	-5.593248000	0.675567000	-3.329085000

5. Transient absorption measurements of ^{3}DPA

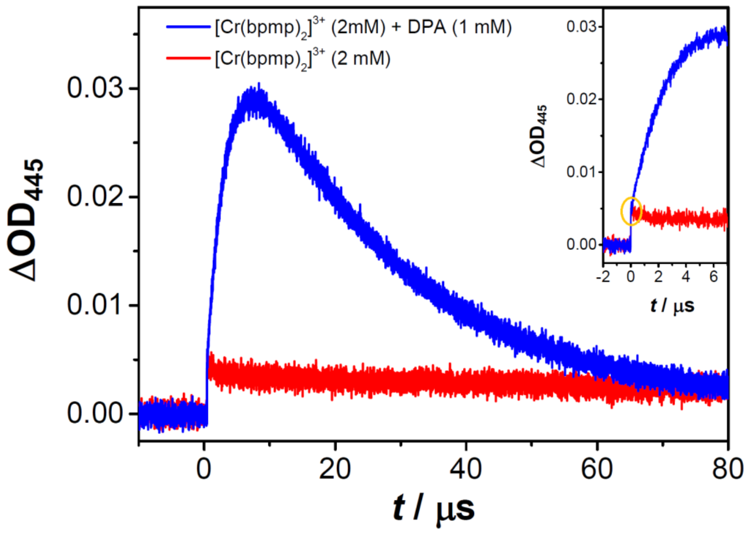

Figure S5: Transient absorption traces upon 532 nm laser excitation (~45 mJ pulse energy) of $[Cr(bpmp)_2][PF_6]_3$ in Ar-saturated acidified DMF monitored at 445 nm. Shown are comparative traces without (red) and with 1 mM DPA (blue). The transient absorption amplitudes directly after the laser pulse are highlighted in the inset, which displays the post-pulse region on enlarged scale. The seemingly static signal increase in the blue curve is due to (weak) $[Cr(bpmp)_2][PF_6]_3$-derived signals and not a result of static ^{3}DPA formation.

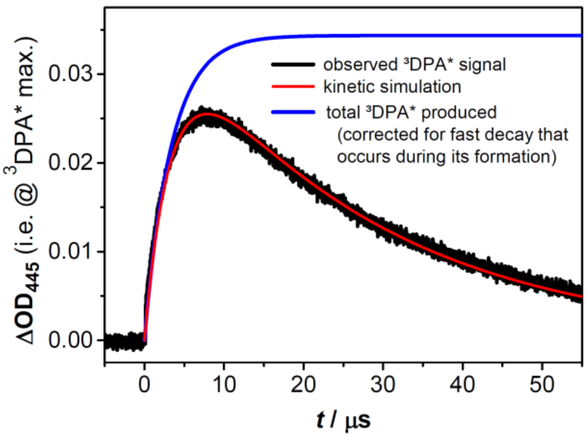

Figure S6: Transient absorption trace from Figure 6a) of the main paper (excitation of $[Cr(bpmp)_2][PF_6]_3$ in the presence of 1 mM DPA with ~40 mJ laser pulses at 532 nm), together with a kinetic simulation allowing the quantification of the ^{3}DPA formation. The experimental trace (black) was simulated with conventional $A \xrightarrow{k1} B \xrightarrow{k2} C$ kinetics with k_1 being dynamic DTET to give ^{3}DPA (1/~5 μs) and k_2 being the ^{3}DPA decay (1/~27 μs) under our conditions. The latter rate constant was obtained by fitting the long-term kinetics (t > 20 μs) individually. This procedure directly yields the maximum ΔOD value of intermediate B in this kinetic model (i.e. ^{3}DPA, see blue curve), as previously described by one of us for a related reaction sequence.[13] Two independent measurements on the maximum ^{3}DPA formation were carried out for both $[Cr(bpmp)_2][PF_6]_3$ and $[Ru(bpy)_3][PF_6]_2$, and they gave practically identical results for the DTET quantum yield (0.92 (92%) ± 0.05, see main paper for details). A systematic error that could potentially arise from very weak contributions of excited $[Cr(bpmp)_2][PF_6]_3$ to the transient absorption signal at 445 nm is expected to be smaller than 5%.

6. Delayed sensitizer phosphorescence measurements

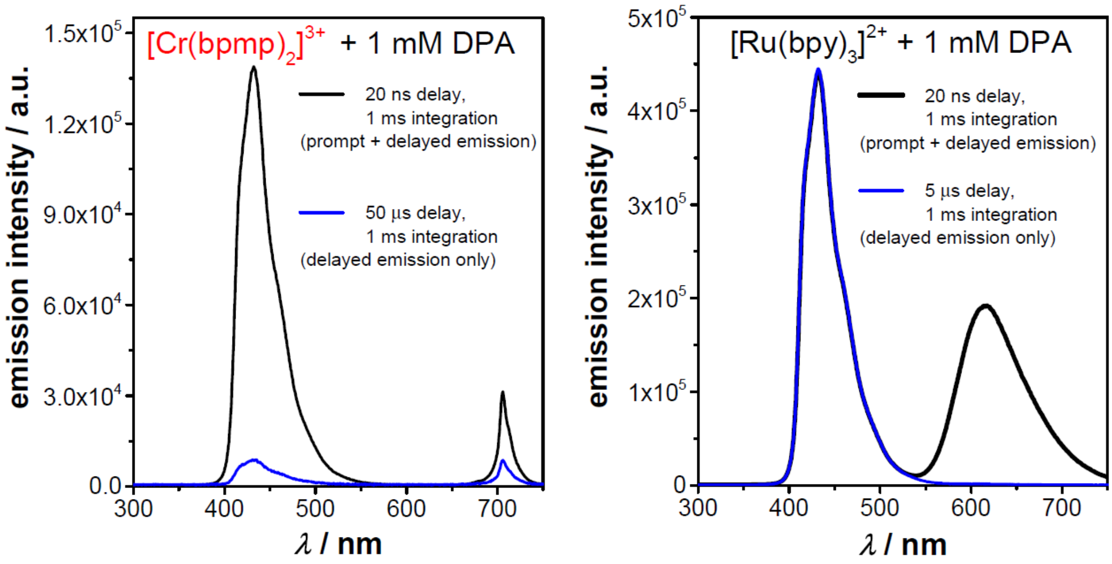

Figure S7: Time-gated emission spectra upon pulsed laser excitation (532 nm, ~5 ns pulse duration, ~40 mJ pulse energy) of Ar-saturated acidified DMF solutions containing DPA (1 mM) and either $[Cr(bpmp)_2][PF_6]_3$ (left) or $[Ru(bpy)_3][PF_6]_2$ (right) as sensitizer under the integration conditions (relative to the laser pulse) as displayed in the respective subfigure. The delayed emission spectra (blue) have been measured with a sufficiently long-time delay that ensures complete decay of the initial (prompt) sensitizer emission.

In contrast to the $[Cr(bpmp)_2]^{3+}$/DPA pair (Figure S7, left), time-gated emission measurements with the $[Ru(bpy)_3]^{2+}$/DPA pair (Figure S7, right) did not yield a detectable delayed phosphorescence of the sensitizer. This is assigned to the large energy difference of the corresponding excited states of the $[Ru(bpy)_3]^{2+}$/DPA couple of about 0.3 eV, which prohibits significant back-EnT.

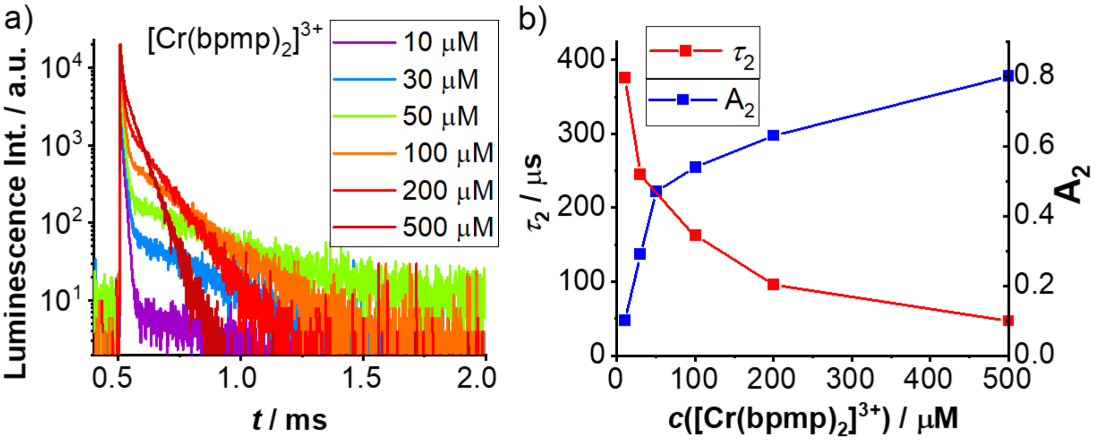

Figure S8: a) Phosphorescence decay kinetics of the sensitizer $[Cr(bpmp)_2][PF_6]_3$ obtained for varying concentrations of the Cr^{III} complex in the presence of 1 mM DPA in deaerated acidified DMF solution. Excitation was at 462 nm using a pulsed xenon lamp (frequency of 100 Hz) and detection at 709 nm, respectively. b) Phosphorescence lifetime τ_2 (delayed phosphorescence emission from TDET) and its relative amplitude obtained from the bi-exponentially fitted decay curves recorded for different $[Cr(bpmp)_2][PF_6]_3$ concentrations.

Table S1: Summary of the phosphorescence decay kinetics fitted bi-exponentially and the corresponding phosphorescence lifetimes τ_{709} and the relative amplitudes of $[Cr(bpmp)_2][PF_6]_3$ at different concentrations in the presence of 1 mM DPA. Excitation was at 462 nm with a flash xenon lamp. Estimated uncertainty of the lifetime is ±5%.

$[Cr(bpmp)_2]^{3+}$ [µM]	τ_1 [µs]	A_1	τ_2 [µs]	A_2
10	8.9	0.90	375	0.10
30	8.8	0.71	245	0.29
50	8.7	0.52	307	0.48
100	8.5	0.46	163	0.54
200	8.4	0.37	96	0.63
500	7.0	0.2	47	0.80

At high $[Cr(bpmp)_2][PF_6]_3$ concentrations (Figure S8, Table S1), τ_2 is shortened while its amplitude A_2 increases. An increasing sensitizer concentration leads to an increasing number of DPA molecules in the triplet state via DTET. This subsequently feeds the TDET process, which accounts for the increase of A_2. The shortened τ_2 of the delayed sensitizer phosphorescence is assigned to the deactivation via DTET, providing a clear hint for the excited state equilibrium[14] between the $^2E/^2T_2$ states of $[Cr(bpmp)_2][PF_6]_3$ and the T_1 state of DPA.

7. Photon upconversion studies of $[Cr(bpmp)_2][PF_6]_3$/DPA pair

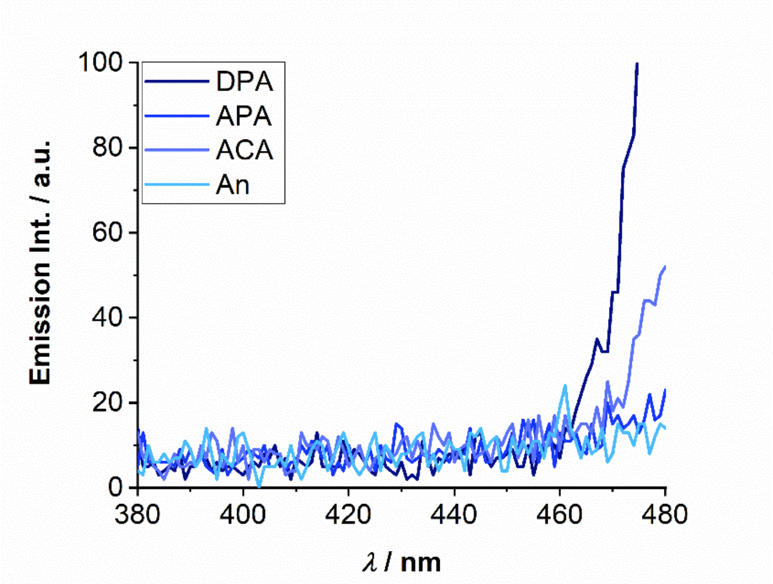

Figure S9: Emission spectra of the anthracene annihilator solutions (1 mM) in acidified DMF under 532 nm (cw, 62 mW) laser excitation in the absence of a sensitizer.

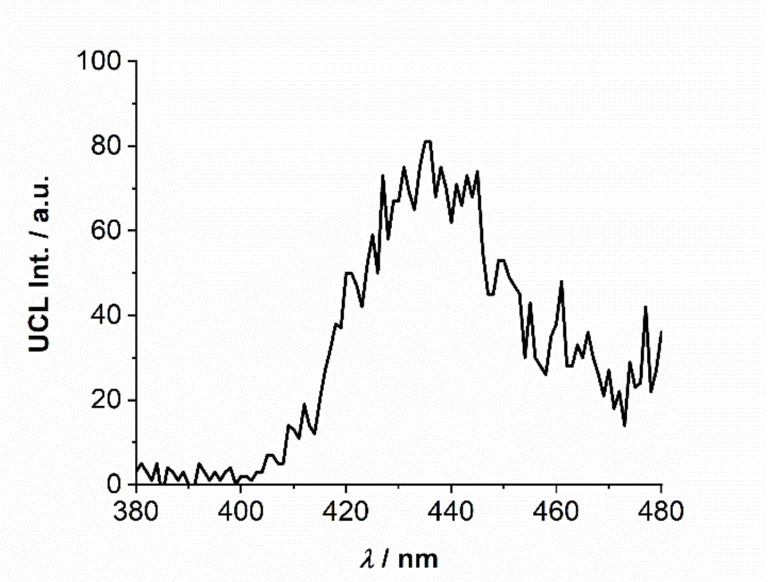

Figure S10: sTTA-UC emission spectrum of $[Cr(bpmp)_2][PF_6]_3$ (50 µM)/DPA (1 mM) in deoxygenated acidified DMF under xenon lamp excitation at 532 nm (excitation power density of ca. 1 mW·cm^{-2}, excitation and emission slits: 12 nm, a 532 nm notch filter was placed between the sample and the detector).

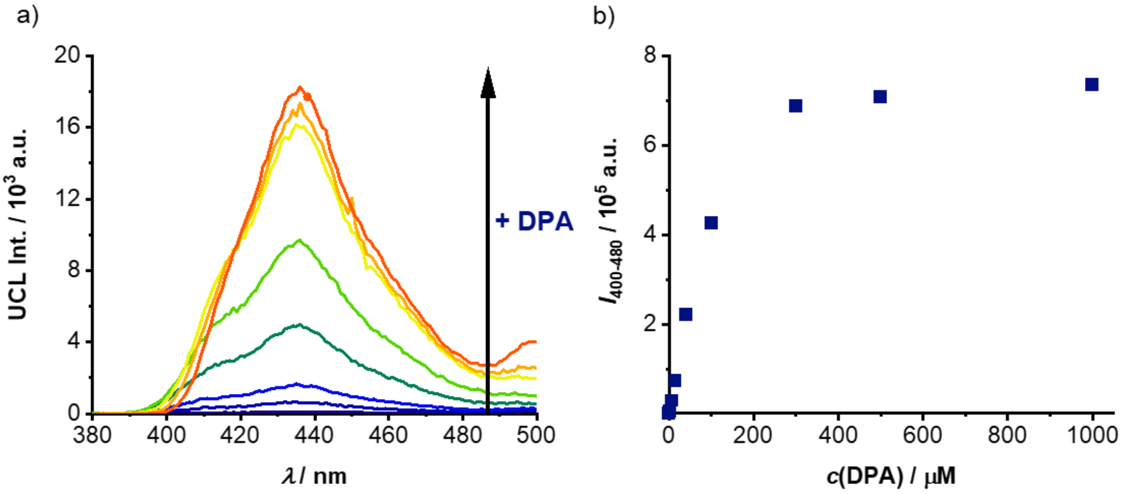

Figure S11: a) sTTA-UC emission spectra of $[Cr(bpmp)_2][PF_6]_3$ (500 µM) obtained with increasing DPA concentration up to 1 mM; b) Plot of the integrated UCL emission intensity as a function of DPA concentration. Excitation with a 532 nm laser (cw, 7 mW).

Power density dependence and Φ_{UC} determination

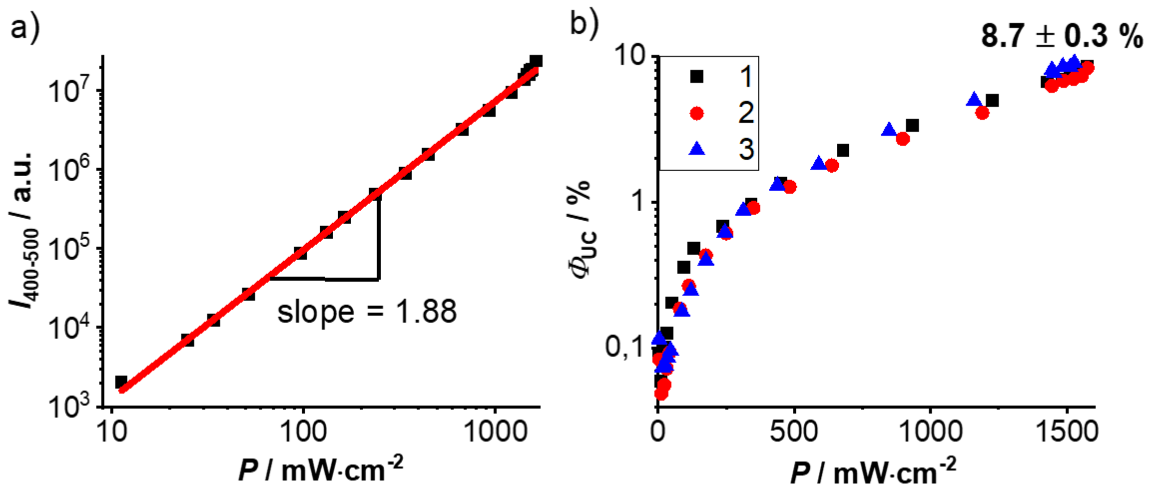

Figure S12: a) Excitation power density dependence of the integrated UCL ($I_{400-500}$) of DPA from the $[Cr(bpmp)_2][PF_6]_3$/DPA pair with a 532 nm laser with increasing power density up to ca. 1.5 W·cm^{-2}, linear fit gives a slope of 1.88; b) relatively determined Φ_{UC} of the $[Cr(bpmp)_2][PF_6]_3$/DPA pair as function of excitation power density of the 532 nm laser according to three independent sets of measurements.

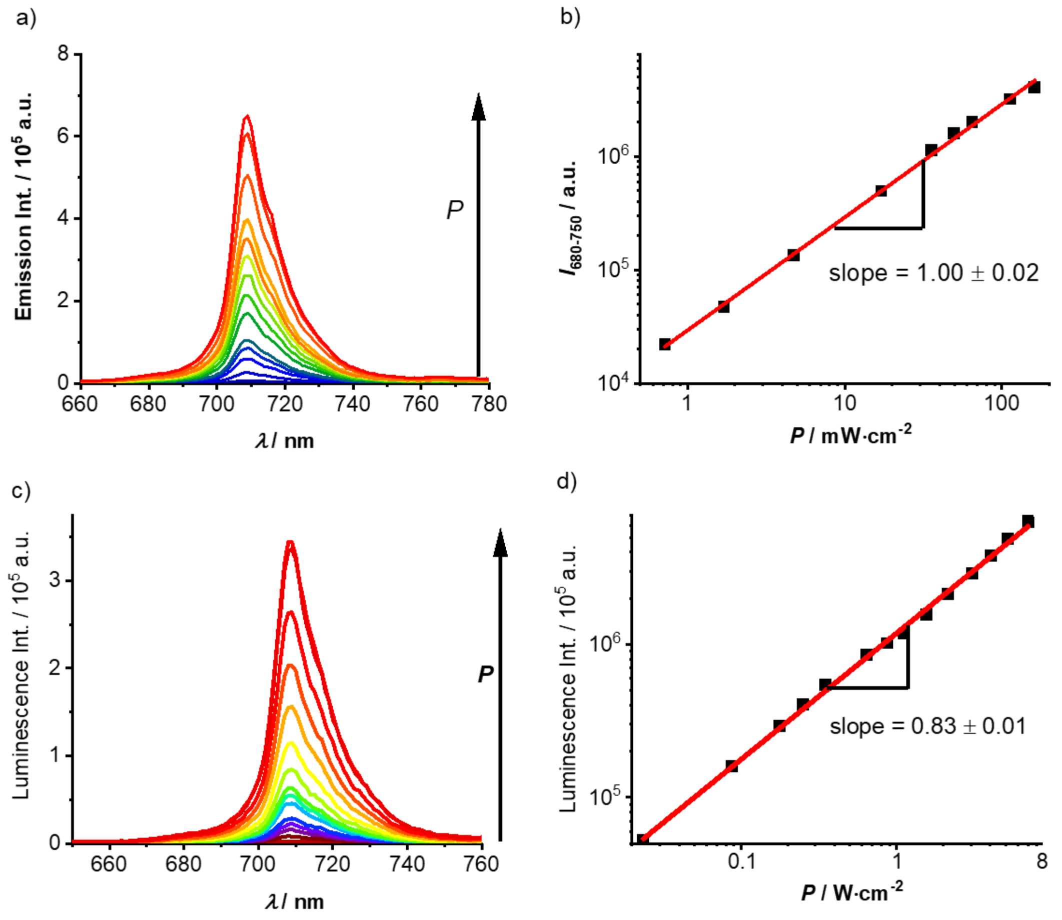

Figure S13*:* a) Phosphorescence spectra of the reference solution $[Cr(bpmp)_2][PF_6]_3$ (50 µM) in acidified DMF with increasing excitation power density of a 532 nm laser (cw, 1.5 W·cm^{-2}), b) excitation power density dependence of integrated the phosphorescence intensity ($I_{680-750}$) of $[Cr(bpmp)_2][PF_6]_3$; linear fit gives a slope of ca. 1. c) Phosphorescence spectra of the reference solution $[Cr(bpmp)_2][PF_6]_3$ (50 µM) in deoxygenated acidified DMF measured with increasing excitation power density of a 520 nm laser (cw, 8 W·cm^{-2}), d) excitation power density dependence of the integrated phosphorescence intensity ($I_{680-750}$) of $[Cr(bpmp)_2][PF_6]_3$; the linear fit gives a slope of ca. 0.83.

Photostability

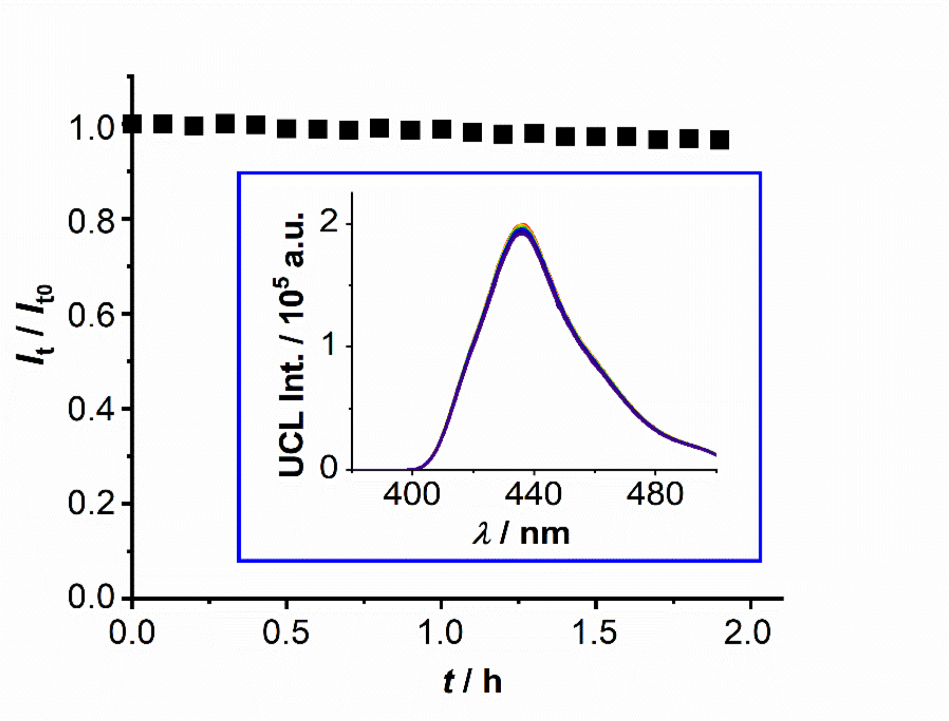

Figure S14: Photostability study of $[Cr(bpmp)_2][PF_6]_3$ (50 µM)/DPA (1 mM) in deaerated acidified DMF under laser (532 nm, cw, 1.5 W·cm^{-2}) illumination for 2 hours, inset: UCL emission spectra were taken every 2 min.

8. Photon upconversion and photodimerization with other anthracenes

Triplet energies of annihilators

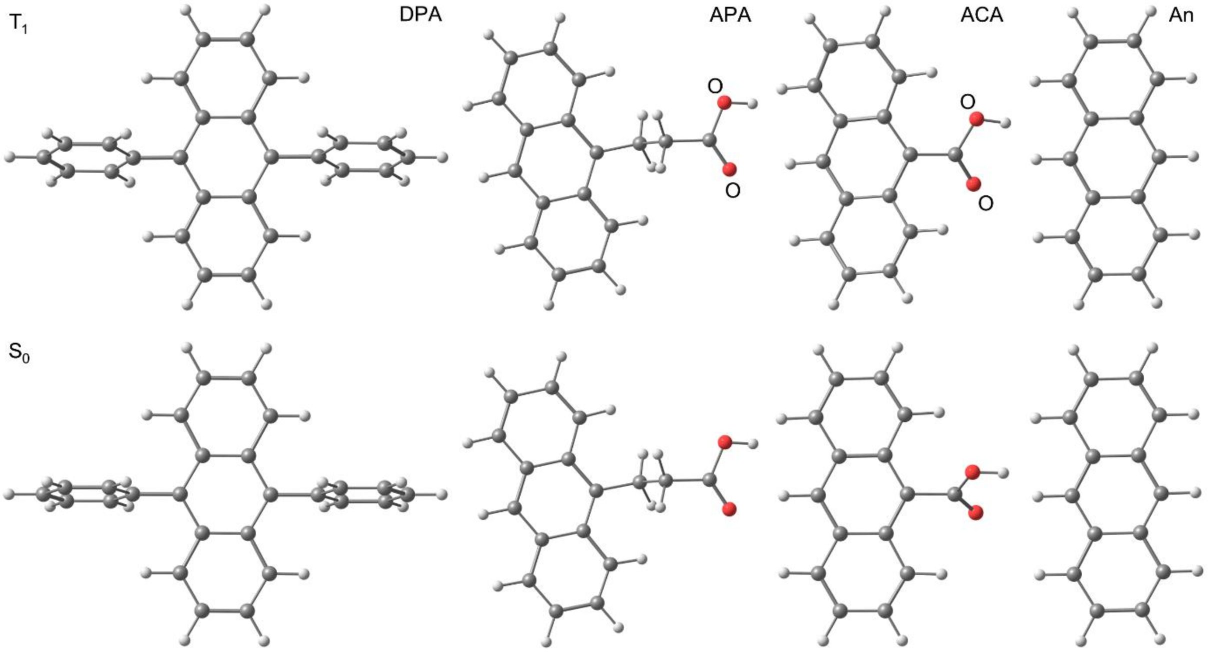

Figure S15*:* Geometries of the anthracene annihilators (DPA, APA, ACA, and An) in the first triplet state (T_1) and ground state (S_0) optimized at the CPCM(DMF)-RI-B3LYP-D3BJ-ZORA/def2-TZVPP basis of theory.

DPA, S_0; CPCM(DMF)-RI-B3LYP-D3BJ-ZORA/def2-TZVPP

–27261.3900 eV

6	1.398963000	3.060850000	12.714689000
6	0.146327000	2.792054000	13.345514000
1	0.057996000	1.914961000	13.968623000
6	-0.926958000	3.614291000	13.171706000
1	-1.866452000	3.385829000	13.657187000
6	-0.817851000	4.771778000	12.357060000
1	-1.676318000	5.417343000	12.227551000
6	0.361578000	5.070612000	11.739236000
1	0.440483000	5.955748000	11.125667000
6	1.510115000	4.234769000	11.886291000
6	2.729572000	4.533784000	11.257382000
6	2.860742000	5.761922000	10.425214000
6	2.424835000	5.788242000	9.100042000
1	1.982371000	4.900526000	8.666918000
6	2.555786000	6.943684000	8.336099000
1	2.211176000	6.950613000	7.310466000
6	3.124892000	8.086797000	8.888293000
1	3.227281000	8.984432000	8.292979000
6	3.564279000	8.068194000	10.208256000
1	4.009589000	8.951523000	10.646816000
6	3.433282000	6.912568000	10.969408000
1	3.779159000	6.898097000	11.994507000
6	3.838455000	3.692025000	11.436848000
6	5.090957000	3.960540000	10.805644000
1	5.179231000	4.837454000	10.182279000
6	6.164220000	3.138264000	10.979399000
1	7.103609000	3.366518000	10.493618000
6	6.055227000	1.981016000	11.794396000
1	6.913686000	1.335449000	11.923939000
6	4.875876000	1.682387000	12.412464000
1	4.797011000	0.797387000	13.026237000
6	3.727317000	2.518192000	12.265323000
6	2.507876000	2.219137000	12.894222000
6	2.377029000	0.991306000	13.726897000
6	2.811188000	0.966548000	15.052675000
1	3.251917000	1.855173000	15.485717000
6	2.680716000	-0.188489000	15.817302000
1	3.023957000	-0.194228000	16.843404000
6	2.113797000	-1.332719000	15.265175000
1	2.011790000	-2.230060000	15.860996000
6	1.676100000	-1.315645000	13.944625000
1	1.232456000	-2.199853000	13.506148000
6	1.806632000	-0.160416000	13.182787000
1	1.462053000	-0.147101000	12.157237000

DPA, T_1; CPCM(DMF)-RI-B3LYP-D3BJ-ZORA/def2-TZVPP

−27259.7599 eV

6	1.346732000	3.044691000	12.664491000
6	0.113976000	2.774721000	13.263780000
1	0.022703000	1.919304000	13.914826000
6	-1.007717000	3.588885000	13.045230000
1	-1.948639000	3.343471000	13.518876000
6	-0.897255000	4.701315000	12.238953000
1	-1.751670000	5.342184000	12.068189000
6	0.336538000	5.009128000	11.642599000
1	0.418970000	5.895677000	11.032965000
6	1.462720000	4.202301000	11.825519000
6	2.736082000	4.529877000	11.239989000
6	2.871589000	5.749598000	10.416340000
6	2.264526000	5.850719000	9.160273000
1	1.704592000	5.011337000	8.770080000
6	2.382072000	7.013648000	8.407286000
1	1.910189000	7.073761000	7.435457000
6	3.098811000	8.099412000	8.901854000
1	3.187919000	9.004637000	8.316170000
6	3.710110000	8.008688000	10.149165000
1	4.270505000	8.846371000	10.542751000
6	3.601273000	6.842381000	10.895902000
1	4.075395000	6.778289000	11.866039000
6	3.891173000	3.708462000	11.487503000
6	5.123955000	3.978485000	10.888300000
1	5.215218000	4.833882000	10.237229000
6	6.245684000	3.164409000	11.106985000
1	7.186628000	3.409867000	10.633404000
6	6.135244000	2.052021000	11.913325000
1	6.989697000	1.411233000	12.084204000
6	4.901428000	1.744148000	12.509594000
1	4.819000000	0.857631000	13.119276000
6	3.775201000	2.550871000	12.326489000
6	2.501802000	2.223209000	12.911907000
6	2.366262000	1.003533000	13.735617000
6	2.973045000	0.902611000	14.991834000
1	3.532830000	1.742085000	15.382042000
6	2.855347000	-0.260195000	15.744983000
1	3.326990000	-0.320130000	16.716938000
6	2.138746000	-1.346053000	15.250425000
1	2.049514000	-2.251182000	15.836237000
6	1.527741000	-1.255555000	14.002958000
1	0.967451000	-2.093308000	13.609377000
6	1.636756000	-0.089373000	13.256061000
1	1.162819000	-0.025542000	12.285812000

APA, S_0; CPCM(DMF)-RI-B3LYP-D3BJ-ZORA/def2-TZVPP

−21960.3503 eV

6	3.648319000	0.459001000	0.059291000
6	3.598951000	-0.959204000	0.108513000
6	2.395098000	-1.598211000	0.076560000
6	1.174232000	-0.864917000	-0.006952000
6	2.501931000	1.195298000	-0.030196000
6	1.216914000	0.573086000	-0.072464000
6	-0.056714000	-1.515616000	-0.021068000
6	-1.251224000	-0.802408000	-0.082802000
6	0.017958000	1.309355000	-0.161821000
6	-1.219745000	0.637608000	-0.146343000
6	-2.509202000	-1.474316000	-0.075119000
6	-3.680183000	-0.777173000	-0.112008000
6	-3.657900000	0.642459000	-0.157766000
6	-2.473948000	1.321958000	-0.177573000
1	4.606246000	0.960988000	0.094842000
1	4.517842000	-1.525831000	0.177331000
1	2.341197000	-2.678970000	0.120883000
1	2.577639000	2.271817000	-0.060492000
1	-0.086671000	-2.597665000	0.028551000
1	-2.511238000	-2.556541000	-0.032573000
1	-4.628034000	-1.298666000	-0.102074000
1	-4.590993000	1.189914000	-0.178730000
1	-2.492159000	2.401030000	-0.210442000
6	0.077733000	2.813538000	-0.238327000
1	0.941340000	3.117274000	-0.826506000
6	0.175990000	3.458479000	1.162623000
1	-0.791381000	3.210503000	-0.756185000
1	0.985248000	2.993630000	1.721886000
1	-0.754097000	3.312285000	1.707617000
6	0.490642000	4.922577000	1.043131000
8	1.603672000	5.371396000	0.871659000
8	-0.603548000	5.700159000	1.100087000
1	-0.336879000	6.624591000	0.971484000

APA, T_1; CPCM(DMF)-RI-B3LYP-D3BJ-ZORA/def2-TZVPP

−21958.7113 eV

6	3.719021000	0.374202000	0.060363000
6	3.644914000	-1.001618000	0.098251000
6	2.387634000	-1.627841000	0.062524000
6	1.209025000	-0.882010000	-0.014554000
6	2.541801000	1.144511000	-0.023484000
6	1.279739000	0.551772000	-0.068327000
6	-0.062449000	-1.512188000	-0.038164000
6	-1.275299000	-0.773315000	-0.082255000
6	0.060781000	1.319435000	-0.163924000
6	-1.222900000	0.662247000	-0.132724000
6	-2.513561000	-1.417258000	-0.070759000
6	-3.715152000	-0.687957000	-0.095911000
6	-3.672709000	0.689382000	-0.126373000
6	-2.432598000	1.358985000	-0.145591000
1	4.677502000	0.874307000	0.094233000
1	4.543435000	-1.600292000	0.161442000
1	2.319561000	-2.707914000	0.101080000
1	2.638242000	2.219418000	-0.052701000
1	-0.111611000	-2.593287000	0.003410000
1	-2.538759000	-2.499297000	-0.035974000
1	-4.662607000	-1.209860000	-0.086359000
1	-4.586771000	1.267480000	-0.140169000
1	-2.435658000	2.438330000	-0.171675000
6	0.139208000	2.813317000	-0.239433000
1	1.006327000	3.112981000	-0.827210000
6	0.246920000	3.455133000	1.168341000
1	-0.729535000	3.220402000	-0.750589000
1	1.128144000	3.075715000	1.679123000
1	-0.636039000	3.201582000	1.752843000
6	0.370957000	4.948809000	1.067295000
8	1.414969000	5.564587000	1.061569000
8	-0.822517000	5.552072000	0.931658000
1	-0.682978000	6.505595000	0.819067000

ACA, S_0; CPCM(DMF)-RI-B3LYP-D3BJ-ZORA/def2-TZVPP

−19821.1873 eV

6	-1.170130000	-0.706193000	5.552378000
1	-1.407237000	-0.136694000	6.437929000
6	-1.466049000	-2.038941000	5.517240000
1	-1.934744000	-2.506299000	6.373286000
6	-1.171829000	-2.824845000	4.372407000
1	-1.415942000	-3.878825000	4.368460000
6	-0.596172000	-2.245234000	3.281732000
1	-0.372915000	-2.827931000	2.396769000
6	-0.278388000	-0.855451000	3.272707000
6	1.157794000	1.699085000	0.952261000
1	1.347046000	1.070850000	0.091327000
6	1.463190000	3.027204000	0.925321000
1	1.893283000	3.473795000	0.038828000
6	1.226645000	3.831308000	2.070574000
1	1.488305000	4.880971000	2.046513000
6	0.681357000	3.298338000	3.201929000
1	0.513409000	3.932091000	4.058836000
6	0.322478000	1.917830000	3.268336000
6	-0.240893000	1.316478000	4.411187000
6	-0.554267000	-0.057551000	4.439129000
6	0.285418000	-0.257283000	2.148761000
1	0.493572000	-0.863712000	1.275497000
6	0.587322000	1.101524000	2.114062000
6	-0.530153000	2.181461000	5.591109000
8	0.062752000	1.751627000	6.715245000
1	-0.194764000	2.339019000	7.444284000
8	-1.206912000	3.186763000	5.560601000

ACA, T_1; CPCM(DMF)-RI-B3LYP-D3BJ-ZORA/def2-TZVPP

−19819.5630 eV

6	-1.162514000	-0.711037000	5.542608000
1	-1.398969000	-0.142316000	6.428889000
6	-1.477102000	-2.082337000	5.524569000
1	-1.947611000	-2.531140000	6.389271000
6	-1.187071000	-2.843564000	4.412656000
1	-1.425884000	-3.898890000	4.390508000
6	-0.589844000	-2.238692000	3.296932000
1	-0.365337000	-2.829183000	2.417083000
6	-0.278533000	-0.876971000	3.288704000
6	1.139715000	1.709960000	0.954472000
1	1.323698000	1.087761000	0.087506000
6	1.466752000	3.073947000	0.916845000
1	1.893274000	3.501949000	0.019089000
6	1.250800000	3.857122000	2.030363000
1	1.512487000	4.906890000	2.024839000
6	0.691273000	3.291982000	3.189153000
1	0.532816000	3.925603000	4.048140000
6	0.332841000	1.944308000	3.245069000
6	-0.231086000	1.323929000	4.422563000
6	-0.552874000	-0.086453000	4.453633000
6	0.287815000	-0.261463000	2.141324000
1	0.495596000	-0.868076000	1.268884000
6	0.587107000	1.126379000	2.096507000
6	-0.518464000	2.181711000	5.591665000
8	0.005178000	1.714754000	6.738531000
1	-0.257328000	2.310210000	7.458943000
8	-1.144560000	3.223000000	5.547962000

An, S_0; CPCM(DMF)-RI-B3LYP-D3BJ-ZORA/def2-TZVPP

−14684.4691 eV

6	3.667604000	0.583209000	-0.000121000
6	3.618735000	-0.838964000	-0.000129000
6	2.419522000	-1.490067000	-0.000131000
6	1.192484000	-0.762629000	-0.000167000
6	2.516149000	1.316036000	-0.000104000
6	1.242070000	0.676583000	-0.000134000
6	-0.048408000	-1.400428000	-0.000067000
6	-1.242068000	-0.676563000	0.000071000
6	0.048403000	1.400452000	-0.000002000
6	-1.192485000	0.762645000	0.000102000
6	-2.516136000	-1.316038000	0.000100000
6	-3.667595000	-0.583223000	0.000171000
6	-3.618741000	0.838953000	0.000163000
6	-2.419537000	1.490071000	0.000115000
1	4.628264000	1.081079000	0.000163000
1	4.542234000	-1.402656000	0.000310000
1	2.378464000	-2.572409000	0.000030000
1	2.551590000	2.398526000	0.000028000
1	-0.087217000	-2.483577000	-0.000076000
1	0.087199000	2.483598000	0.000033000
1	-2.551545000	-2.398529000	-0.000014000
1	-4.628250000	-1.081104000	-0.000064000
1	-4.542254000	1.402623000	-0.000227000
1	-2.378491000	2.572413000	-0.000049000

An, T_1; CPCM(DMF)-RI-B3LYP-D3BJ-ZORA/def2-TZVPP

−14682.7957 eV

6	3.711380000	0.559528000	-0.000134000
6	3.663981000	-0.818497000	-0.000118000
6	2.419496000	-1.478945000	-0.000178000
6	1.222590000	-0.761925000	-0.000180000
6	2.515132000	1.304978000	-0.000207000
6	1.272197000	0.673469000	-0.000175000
6	-0.048361000	-1.403363000	-0.000053000
6	-1.272194000	-0.673458000	0.000119000
6	0.048361000	1.403369000	-0.000009000
6	-1.222597000	0.761932000	0.000146000
6	-2.515124000	-1.304973000	0.000163000
6	-3.711368000	-0.559532000	0.000150000
6	-3.663996000	0.818493000	0.000173000
6	-2.419509000	1.478949000	0.000206000
1	4.661937000	1.075713000	0.000274000
1	4.576131000	-1.399744000	0.000413000
1	2.384754000	-2.561405000	-0.000055000
1	2.556797000	2.387113000	-0.000131000
1	-0.087254000	-2.485801000	-0.000072000
1	0.087253000	2.485806000	0.000017000
1	-2.556780000	-2.387109000	0.000071000
1	-4.661919000	-1.075731000	-0.000232000
1	-4.576154000	1.399727000	-0.000299000
1	-2.384762000	2.561407000	0.00011300

Stern-Volmer studies of $[Cr(bpmp)_2]^{3+}$ quenched by APA/ACA/An

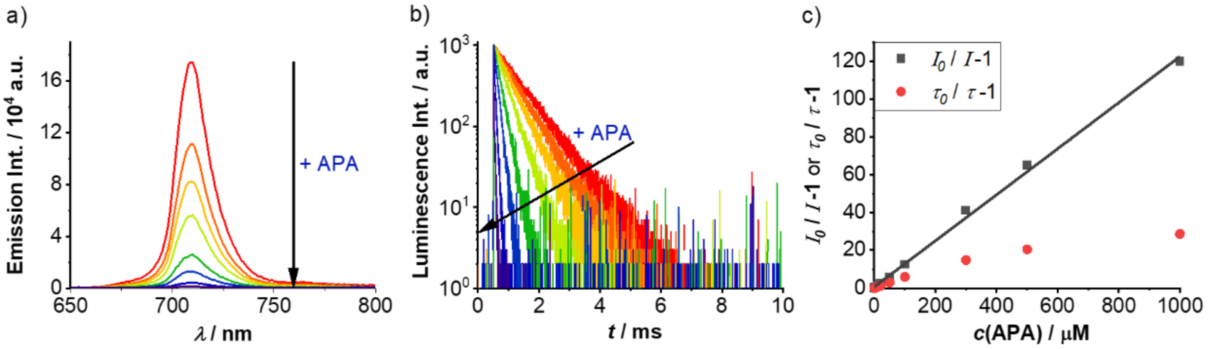

Figure S16: Stern-Volmer study based on a) phosphorescence intensity and b) decay of $[Cr(bpmp)_2][PF_6]_3$ (0.5 mM, in DMF/$HClO_4$) in the presence of APA with different concentrations up to 1 mM (exc. 532 nm, em. 709 nm), and their respective Stern-Volmer plots based on phosphorescence intensity (black) and lifetime (red) with APA concentrations up to 1 mM.

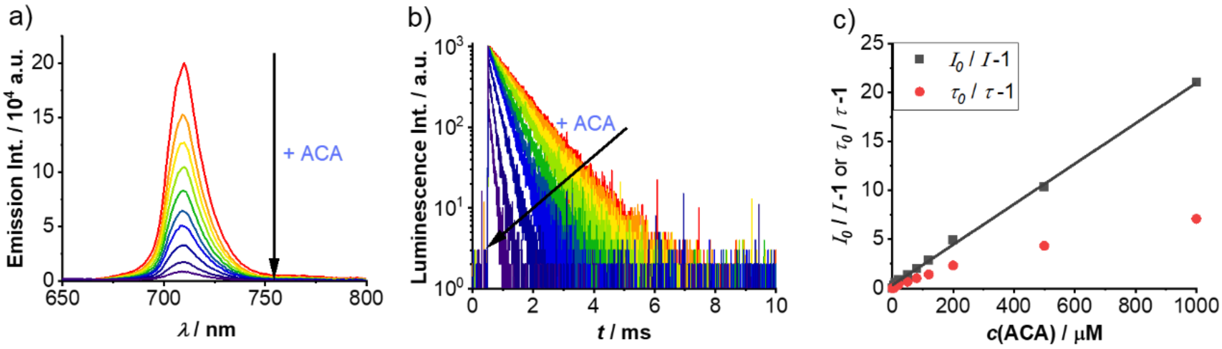

Figure S17: Stern-Volmer study based on a) phosphorescence intensity and b) decay of $[Cr(bpmp)_2][PF_6]_3$ (0.5 mM, in DMF/$HClO_4$) in the presence of ACA with different concentrations (exc. 532 nm, em. 709 nm), and their respective Stern-Volmer plots based on phosphorescence intensity (black) and lifetime (red) with ACA concentrations up to 1 mM.

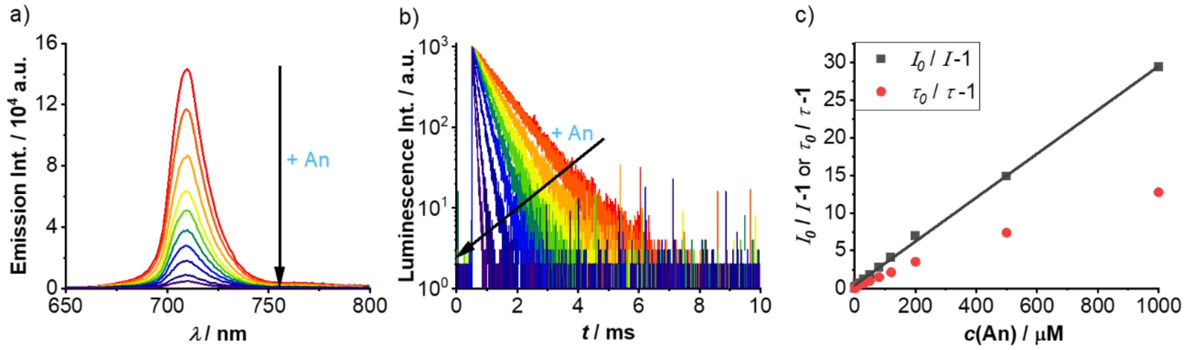

Figure S18: Stern-Volmer study based on a) phosphorescence intensity and b) decay of $[Cr(bpmp)_2][PF_6]_3$ (0.5 mM, in DMF/$HClO_4$) in the presence of An with different concentrations (exc. 532 nm, em. 709 nm), and their respective Stern-Volmer plots based on phosphorescence intensity (black) and lifetime (red) with An concentrations up to 1 mM.

Similar to the $[Cr(bpmp)_2][PF_6]_3$/DPA pair, the non-linear lifetime-based Stern-Volmer plots observed with APA/APA/An annihilators are most likely due to the back-energy transfer from the T_1 state of these anthracenes to the doublet states of the sensitizer.

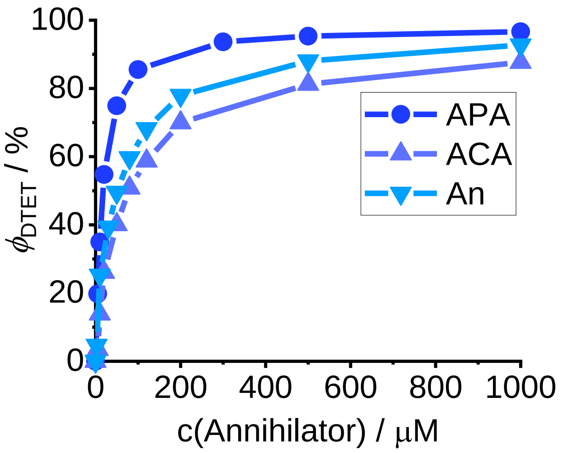

Figure S19: Φ_{DTET} derived from the $[Cr(bpmp)_2][PF_6]_3$ lifetimes plotted as a function of the annihilators (APA/ACA/An) concentration up to 1 mM (exc. 532 nm, em. 709 nm).

Photon upconversion

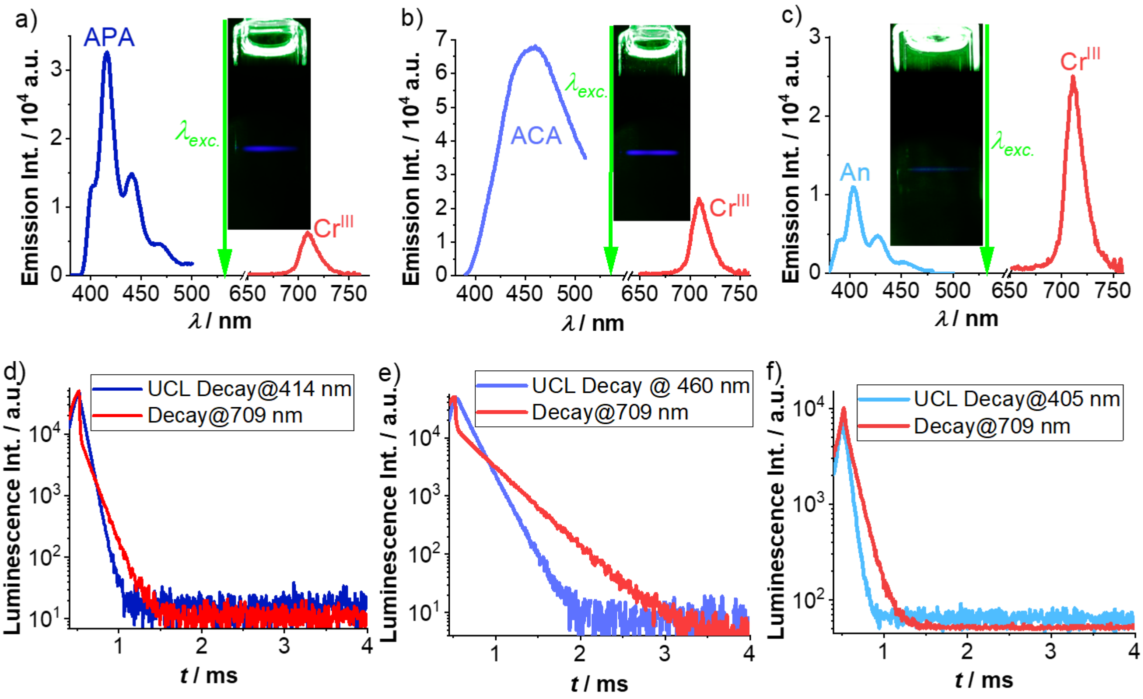

Figure S20: a) UCL spectra and d) UCL decays of $[Cr(bpmp)_2][PF_6]_3$/APA, b) UCL spectra and e) UCL decays of $[Cr(bpmp)_2][PF_6]_3$/ACA, c) UCL spectra and f) UCL decays of $[Cr(bpmp)_2][PF_6]_3$/An, respectively. The red emission traces in d)-f) correspond to the concomitant sensitizer phosphorescence decays. Insets: Photographs of the samples under 532 nm laser excitation (laser power of about 40 mW). The UCL spectra were obtained under 532 nm laser excitation (cw, 1.5 W/cm^2), while the UCL decays were measured with the same laser operated in the pulsed mode (250 Hz, pulse width 500 µs). All samples contain the sensitizer and acceptor concentrations of 50 µM and 1 mM, respectively, in deaerated acidified DMF.

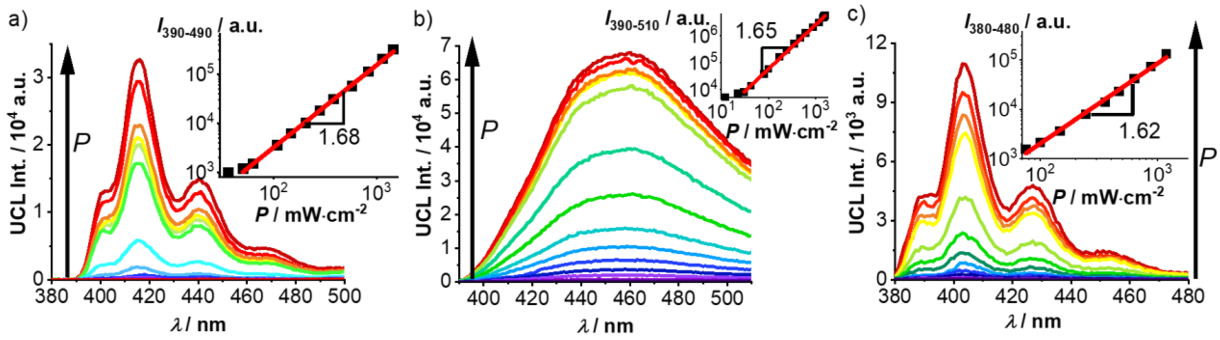

Figure S21: UCL spectra of a) [Cr(bpmp)$_2$][PF$_6$]$_3$/APA, b) [Cr(bpmp)$_2$][PF$_6$]$_3$/ACA, and c) [Cr(bpmp)$_2$][PF$_6$]$_3$/An and integrated UCL as a function of excitation power density (insets) of a 532 nm laser (cw, ca. 1.5 W·cm^{-2}). All UC studies were done with samples containing a sensitizer concentration of 50 µM and an annihilator concentration of 1 mM in deaerated acidified DMF at room temperature.

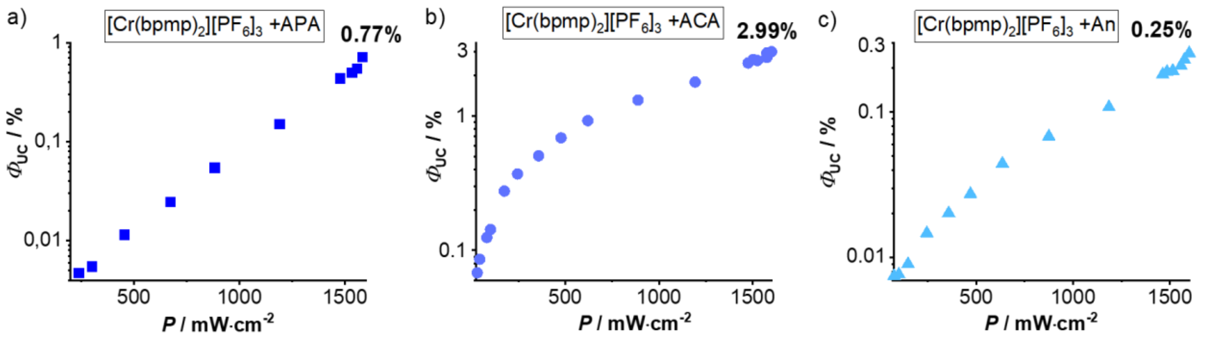

Figure S22: Relatively determined Φ_{UC} of the a) [Cr(bpmp)$_2$][PF$_6$]$_3$/APA, b) [Cr(bpmp)$_2$][PF$_6$]$_3$/ACA, and c) [Cr(bpmp)$_2$][PF$_6$]$_3$/An pairs with increasing power density of the 532 nm-laser up to ca. 1.5 W·cm^{-2}.

Table S2: Summary of the data derived from the SV and UC studies.

Quencher	K_{SV}[a] / 10^4 M^{-1}		k_{DTET}[a] / 10^7 M^{-1}s^{-1}	τ_{UC}[b,d] / µs	τ_{709}[b,d] / µs	Φ_{DTET}[b] / %	Φ_{UC}[b] / %
	based on I_{709}	based on τ_{709}	based on τ_{709}				
**DPA**	5.7	5.0	5.6	162	7 (15%), 303 (85%)	99.2	8.7; 12.0[c]
**APA**	12	5.9	6.7	62	6 (20%), 119 (80%)	99.3	0.77
**ACA**	2.2	1.2	1.4	153	13 (5%), 316 (95%)	98.5	2.99
**An**	3.3	1.8	2.0	57	15 (6%), 113 (94%)	98.3	0.25

[a]: SV studies of [Cr(bpmp)$_2$][PF$_6$]$_3$ (0.5 mM) in deaerated acidified DMF done with increasing concentrations of the four anthracene annihilators. [b]: UC studies of [Cr(bpmp)$_2$][PF$_6$]$_3$ (50 µM) in deaerated acidified DMF in the presence of 1 mM anthracene annihilator. The luminescence decays were obtained with a pulsed 532 nm-laser (250 Hz, pulse width 500 µs). A 532 nm-laser (cw, ca. 1.5 W·cm^{-2}) was used to determine the Φ_{UC} values of all sTTA-UC pairs. [c]: For the [Cr(bpmp)$_2$][PF$_6$]$_3$/DPA pair, a maximum Φ_{UC} value of 12% was obtained with a more intense 520 nm-laser (cw, ca. 8 W·cm^{-2}) enabling to reach saturation. [d]: The delayed fluorescence lifetimes τ_{UC} from the anthracenes were fitted mono-exponentially, while the sensitizer lifetimes τ_{709} were fitted bi-exponentially; the respective relative amplitudes are given in brackets. The estimated uncertainty of the lifetimes amounts to ±5%.

The integrated UCL intensities of APA, ACA, and An showed an excitation power density dependence (532 nm laser, cw, 1.5 W·cm^{-2}) with slopes of about 1.65 (Figure S21), probably due to the involved photodimerization. In addition, the Φ_{UC} values obtained with these annihilators are considerably lower than the [Cr(bpmp)$_2$][PF$_6$]$_3$/DPA pair. The lower Φ_{UC} values are ascribed to the lower Φ_F of the anthracene annihilators (Figure S1), the more efficient TDET due to their higher triplet energies (Figure S15), the inherently less efficient TTA compared to DPA,[15] and the additional deactivation pathway of the UC-activated singlet state of the APA, ACA, and An annihilators via photodimerization (see below).

Photodimerization

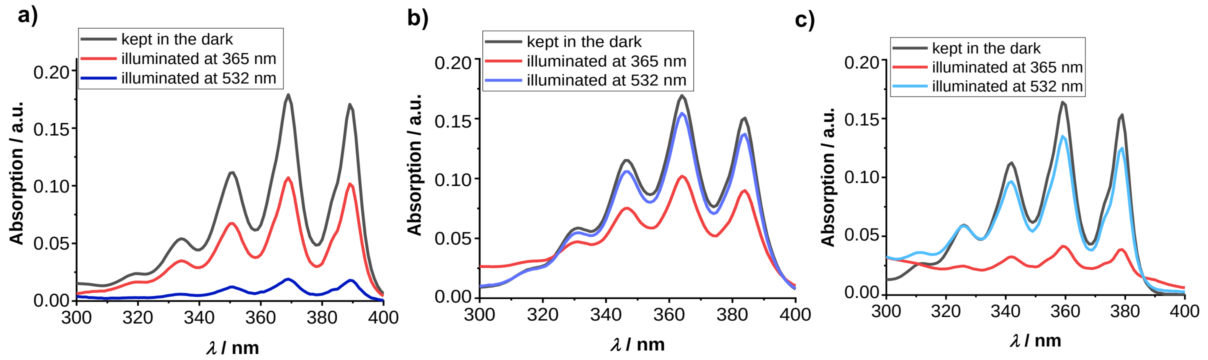

Figure S23: Absorption spectra of a) $[Cr(bpmp)_2][PF_6]_3$/APA, b) $[Cr(bpmp)_2][PF_6]_3$/ACA, and c) $[Cr(bpmp)_2][PF_6]_3$/An kept in the dark under air (black), illuminated with 532 nm laser light without oxygen for 2 hours (blue), and illuminated at 365 nm under air for 10-30 min (red), respectively. All samples were diluted by a factor of 10 for the absorption measurements using an optical cell with a path length of 2 mm.

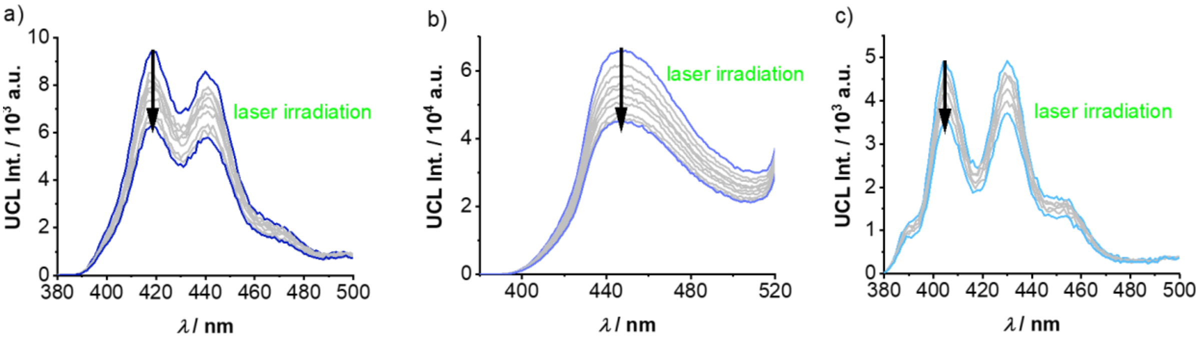

Figure S24: UCL emission spectra of a) $[Cr(bpmp)_2][PF_6]_3$/APA, b) $[Cr(bpmp)_2][PF_6]_3$/ACA, and c) $[Cr(bpmp)_2][PF_6]_3$/An in acidified DMF under laser (532 nm, cw, 1.5 W·cm^{-2}) irradiation up to ca. 1 hour.

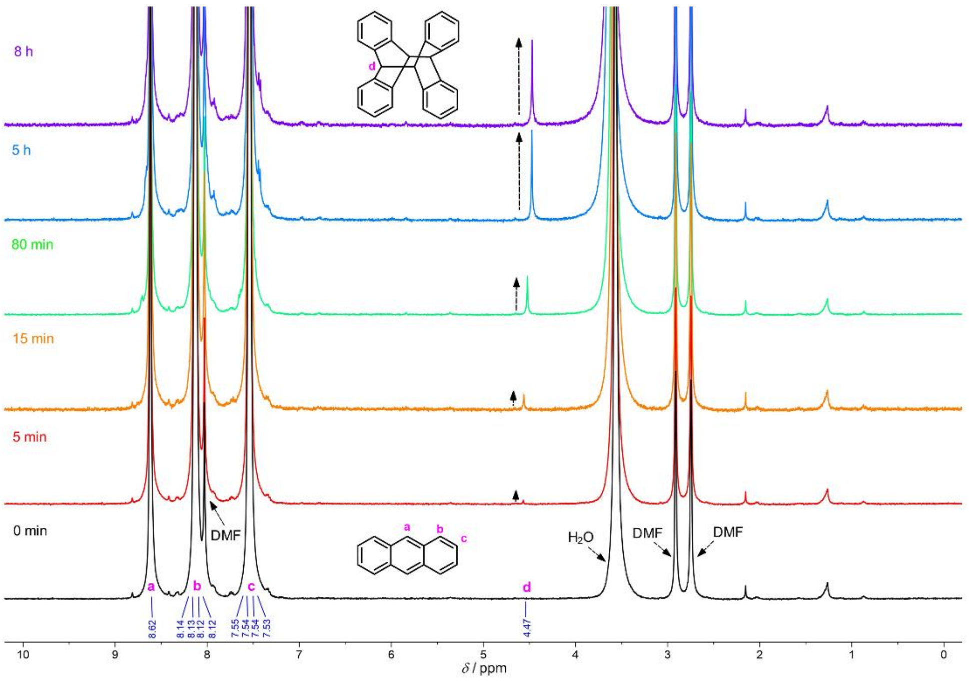

Figure S25: ^{1}H NMR spectra of the solution of the photolysis experiment in d_7-DMF showing the increase of the resonance of the bridgehead protons at 4.47 ppm characteristic for the anthracene dimer.[16]

9. References

[1] F. Reichenauer, C. Wang, C. Förster, P. Boden, N. Ugur, R. Báez-Cruz, J. Kalmbach, L. M. Carrella, E. Rentschler, C. Ramanan, G. Niedner-Schatteburg, M. Gerhards, M. Seitz, U. Resch-Genger,K. Heinze, *J. Am. Chem. Soc.* **2021**, *143*, 11843–11855.

[2] C. Würth, J. Pauli, C. Lochmann, M. Spieles,U. Resch-Genger, *Anal. Chem.* **2012**, *84*, 1345–1352.

[3] a) T. N. Singh-Rachford,F. N. Castellano, *Coord. Chem. Rev.* **2010**, *254*, 2560-2573; b) J. B. Bilger, C. Kerzig, C. B. Larsen,O. S. Wenger, *J. Am. Chem. Soc.* **2021**, *143*, 1651-1663.

[4] Y. Zhou, F. N. Castellano, T. W. Schmidt,K. Hanson, *ACS Energy Lett.* **2020**, *5*, 2322-2326.

[5] a) I. Momiji, C. Yoza,K. Matsui, *J. Phys. Chem. B* **2000**, *104*, 1552-1555

; b) H. Saigusa,E. C. Lim, *Acc. Chem. Res.* **1996**, *29*, 171–178; c) N. S. Bazilevskaya,A. S. Cherkasov, *Opt. Spectrosc.* **1965**, *18*, 30.

[6] F. Neese, *WIREs Comput. Mol. Sci.* **2011**, *2*, 73-78.

[7] a) A. D. Becke, *J. Chem. Phys.* **1993**, *98*, 5648–5652; b) C. Lee, W. Yang,R. G. Parr, *Phys. Rev. B* **1988**, *37*, 785–789; c) B. Miehlich, A. Savin, H. Stoll,H. Preuss, *Chem. Phys. Lett.* **1989**, *157*, 200–206.

[8] a) F. Weigend,R. Ahlrichs, *Phys. Chem. Chem. Phys.* **2005**, *7*, 3297–3305; b) F. Weigend, *Phys. Chem. Chem. Phys.* **2006**, *8*, 1057–1065.

[9] a) F. Neese, F. Wennmohs, A. Hansen,U. Becker, *Chem. Phys.* **2009**, *356*, 98–109; b) R. Izsák,F. Neese, *J. Chem. Phys.* **2011**, *135*, 144105.

[10] a) D. A. Pantazis, X.-Y. Chen, C. R. Landis,F. Neese, *J. Chem. Theory Comput.* **2008**, *4*, 908–919; b) E. van Lenthe, E. J. Baerends,J. G. Snijders, *J. Chem. Phys.* **1993**, *99*, 4597–4610; c) C. van Wüllen, *J. Chem. Phys.* **1998**, *109*, 392–399.

[11] a) S. Grimme, J. Antony, S. Ehrlich,H. Krieg, *J. Chem. Phys.* **2010**, *132*, 154104; b) S. Grimme, S. Ehrlich,L. Goerigk, *J. Comput. Chem.* **2011**, *32*, 1456–1465.

[12] V. Barone,M. Cossi, *J. Phys. Chem. A* **1998**, *102*, 1995–2001.

[13] C. Kerzig,M. Goez, *Phys. Chem. Chem. Phys.* **2014**, *16*, 25342-25349.

[14] D. Meroni, A. Monguzzi,F. Meinardi, *J. Chem. Phys.* **2020**, *153*, 114302.

[15] R. R. Islangulov, D. V. Kozlov,F. N. Castellano, *Chem. Commun.* **2005**, 3776-3778.

[16] S. Baluschev, F. Yu, T. Miteva, S. Ahl, A. Yasuda, G. Nelles, W. Knoll,G. Wegner, *Nano Lett.* **2005**, *5*, 2482-2484.
